# Supplementary material for: Autophagy adaptors mediate Parkin-dependent mitophagy by forming sheet-like liquid condensates
Source: EMBO J. 2024 Oct 17;43(22):5613–34. doi: 10.1038/s44318-024-00272-5 (PMC11574277; doi:10.1038/s44318-024-00272-5)
Supplement: Supplementary file 2 — Movie EV1 [file 44318_2024_272_MOESM2_ESM.zip › Movie EV1/Movie EV1 legend.docx]

**Movie EV1. OPTN condensates redistribute upon mitochondrial contact.**

Time-lapse video of two separate mitochondria approaching and contacting each other (30s per frame). Scale bars indicate 1 μm. See also Figure 4C and D.
