# Supplementary material for: Autophagy adaptors mediate Parkin-dependent mitophagy by forming sheet-like liquid condensates
Source: EMBO J. 2024 Oct 17;43(22):5613–34. doi: 10.1038/s44318-024-00272-5 (PMC11574277; doi:10.1038/s44318-024-00272-5)
Supplement: Supplementary file 3 — Movie EV2 [file 44318_2024_272_MOESM3_ESM.zip › Movie EV2/Movie EV2 legend.docx]

**Movie EV2. OPTN condensates redistribute upon mitochondria–isolation membrane contact.**

Time-lapse video of a mitochondrion with an isolation membrane elongating on its surface (30s per frame). Scale bars indicate 1 μm. See also Figure 4E and F.
