# Supplementary figures and images for: Autophagy adaptors mediate Parkin-dependent mitophagy by forming sheet-like liquid condensates
Source: EMBO J. 2024 Oct 17;43(22):5613–34. doi: 10.1038/s44318-024-00272-5 (PMC11574277; doi:10.1038/s44318-024-00272-5)

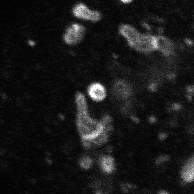

Supplement: Supplementary file 4 — Source data Fig. 1 [file 44318_2024_272_MOESM4_ESM.zip › Figure1/Figure1A/NBR1.tif]

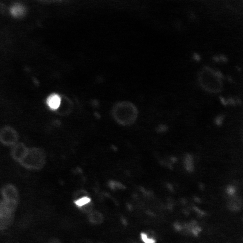

Supplement: Supplementary file 4 — Source data Fig. 1 [file 44318_2024_272_MOESM4_ESM.zip › Figure1/Figure1A/NDP52.tif]

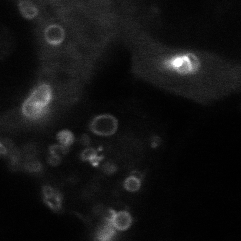

Supplement: Supplementary file 4 — Source data Fig. 1 [file 44318_2024_272_MOESM4_ESM.zip › Figure1/Figure1A/OPTN.tif]

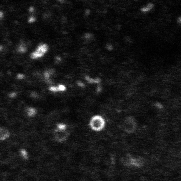

Supplement: Supplementary file 4 — Source data Fig. 1 [file 44318_2024_272_MOESM4_ESM.zip › Figure1/Figure1A/p62.tif]

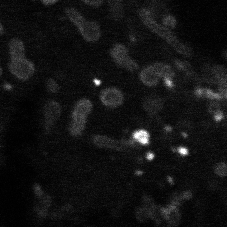

Supplement: Supplementary file 4 — Source data Fig. 1 [file 44318_2024_272_MOESM4_ESM.zip › Figure1/Figure1A/TAX1BP1.tif]

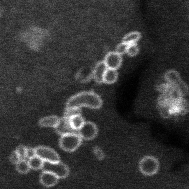

Supplement: Supplementary file 4 — Source data Fig. 1 [file 44318_2024_272_MOESM4_ESM.zip › Figure1/Figure1A/Ub.tif]

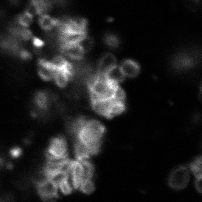

Supplement: Supplementary file 4 — Source data Fig. 1 [file 44318_2024_272_MOESM4_ESM.zip › Figure1/Figure1B/NBR1.tif]

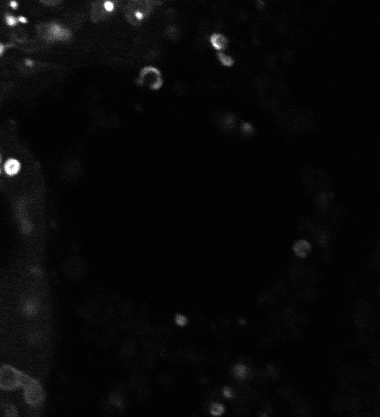

Supplement: Supplementary file 4 — Source data Fig. 1 [file 44318_2024_272_MOESM4_ESM.zip › Figure1/Figure1B/NDP52.tif]

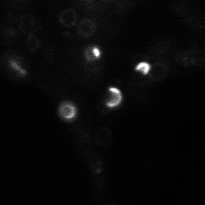

Supplement: Supplementary file 4 — Source data Fig. 1 [file 44318_2024_272_MOESM4_ESM.zip › Figure1/Figure1B/OPTN.tif]

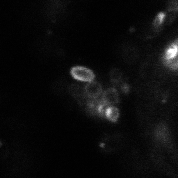

Supplement: Supplementary file 4 — Source data Fig. 1 [file 44318_2024_272_MOESM4_ESM.zip › Figure1/Figure1B/p62.tif]

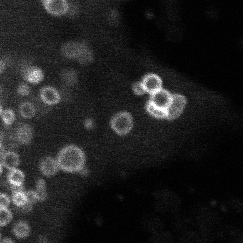

Supplement: Supplementary file 4 — Source data Fig. 1 [file 44318_2024_272_MOESM4_ESM.zip › Figure1/Figure1B/TAX1BP1.tif]

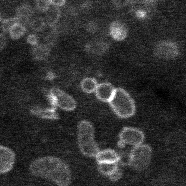

Supplement: Supplementary file 4 — Source data Fig. 1 [file 44318_2024_272_MOESM4_ESM.zip › Figure1/Figure1B/Ub.tif]

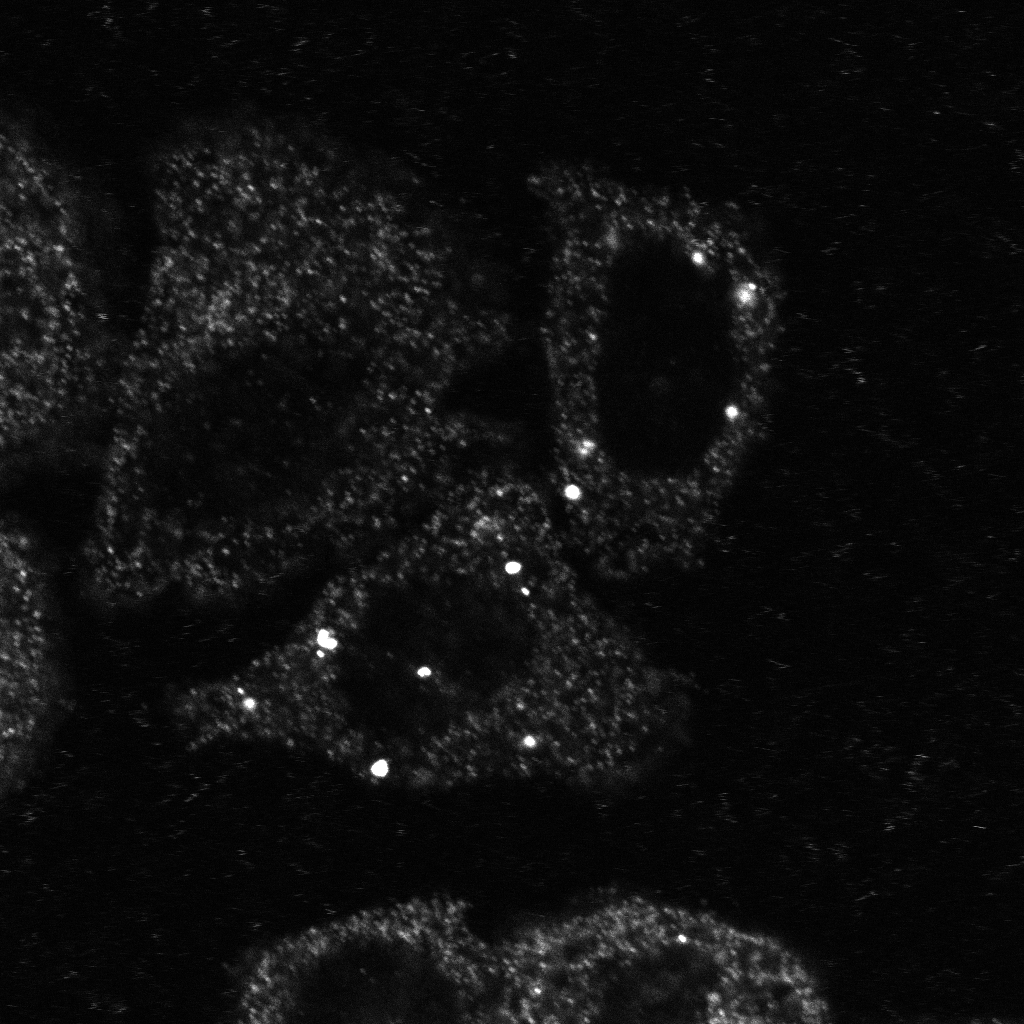

Supplement: Supplementary file 4 — Source data Fig. 1 [file 44318_2024_272_MOESM4_ESM.zip › Figure1/Figure1D/Anti-NDP52.tif]

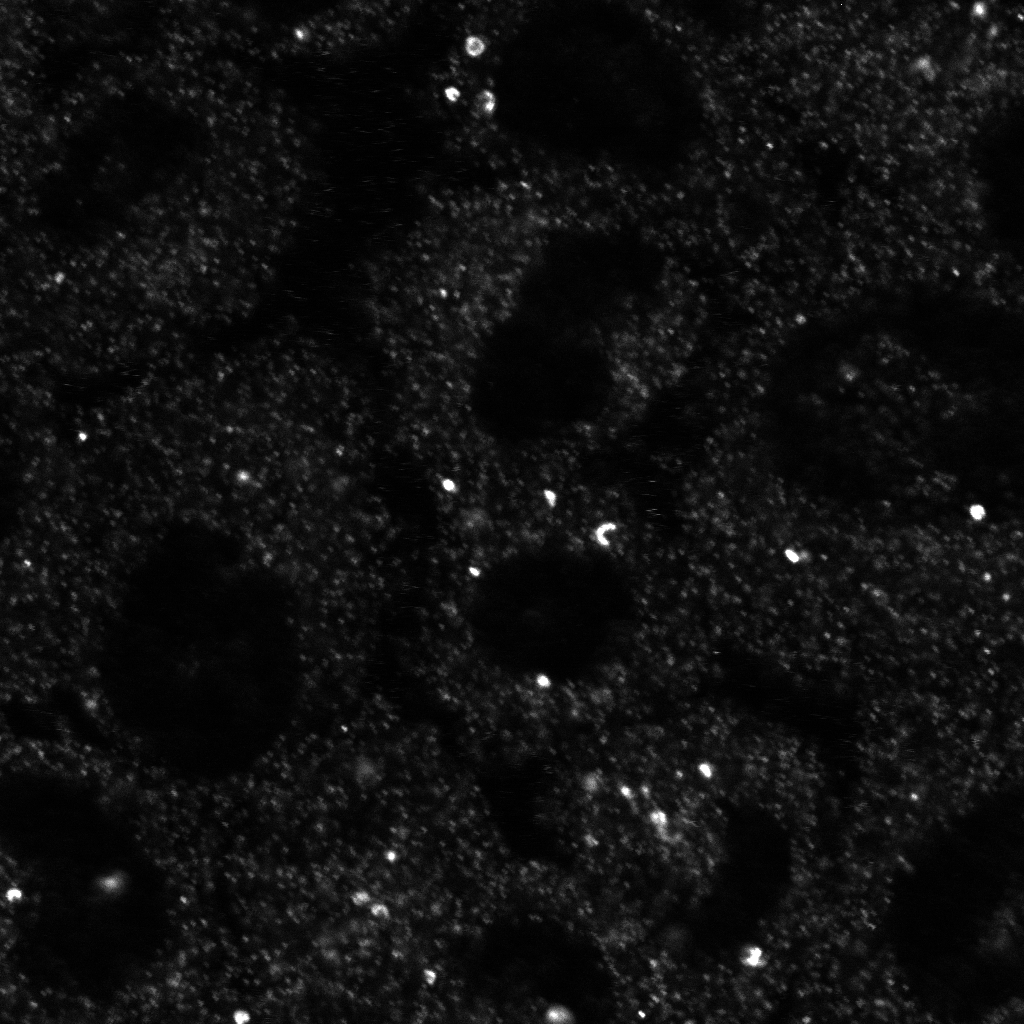

Supplement: Supplementary file 4 — Source data Fig. 1 [file 44318_2024_272_MOESM4_ESM.zip › Figure1/Figure1D/Anti-OPTN.tif]

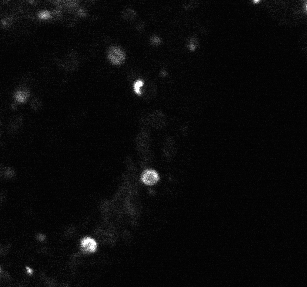

Supplement: Supplementary file 4 — Source data Fig. 1 [file 44318_2024_272_MOESM4_ESM.zip › Figure1/Figure1E/NDP52.tif]

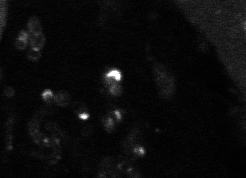

Supplement: Supplementary file 4 — Source data Fig. 1 [file 44318_2024_272_MOESM4_ESM.zip › Figure1/Figure1E/OPTN.tif]

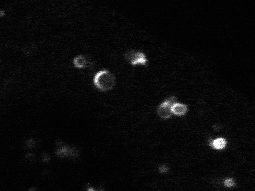

Supplement: Supplementary file 4 — Source data Fig. 1 [file 44318_2024_272_MOESM4_ESM.zip › Figure1/Figure1E/TAX1BP1.tif]

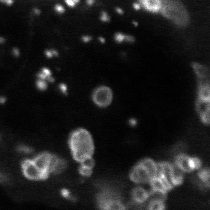

Supplement: Supplementary file 5 — Source data Fig. 2 [file 44318_2024_272_MOESM5_ESM.zip › Figure2/Figure2A/NDP52.tif]

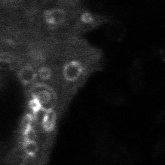

Supplement: Supplementary file 5 — Source data Fig. 2 [file 44318_2024_272_MOESM5_ESM.zip › Figure2/Figure2A/OPTN.tif]

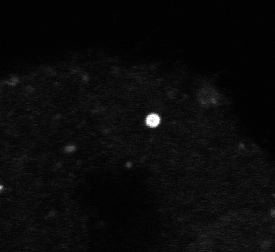

Supplement: Supplementary file 5 — Source data Fig. 2 [file 44318_2024_272_MOESM5_ESM.zip › Figure2/Figure2A/p62.tif]

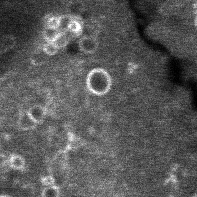

Supplement: Supplementary file 5 — Source data Fig. 2 [file 44318_2024_272_MOESM5_ESM.zip › Figure2/Figure2A/Ub.tif]

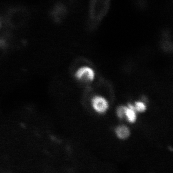

Supplement: Supplementary file 5 — Source data Fig. 2 [file 44318_2024_272_MOESM5_ESM.zip › Figure2/Figure2C/NDP52.tif]

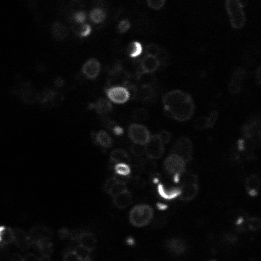

Supplement: Supplementary file 5 — Source data Fig. 2 [file 44318_2024_272_MOESM5_ESM.zip › Figure2/Figure2C/OPTN.tif]

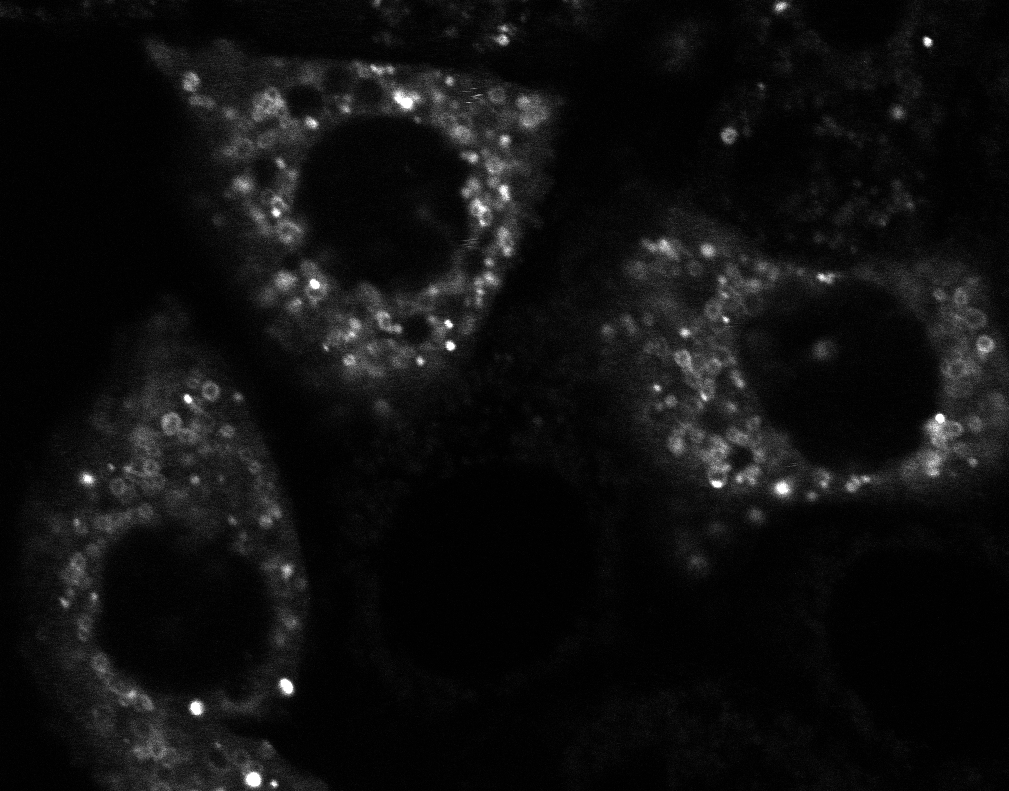

Supplement: Supplementary file 5 — Source data Fig. 2 [file 44318_2024_272_MOESM5_ESM.zip › Figure2/Figure2E/ndp52.tif]

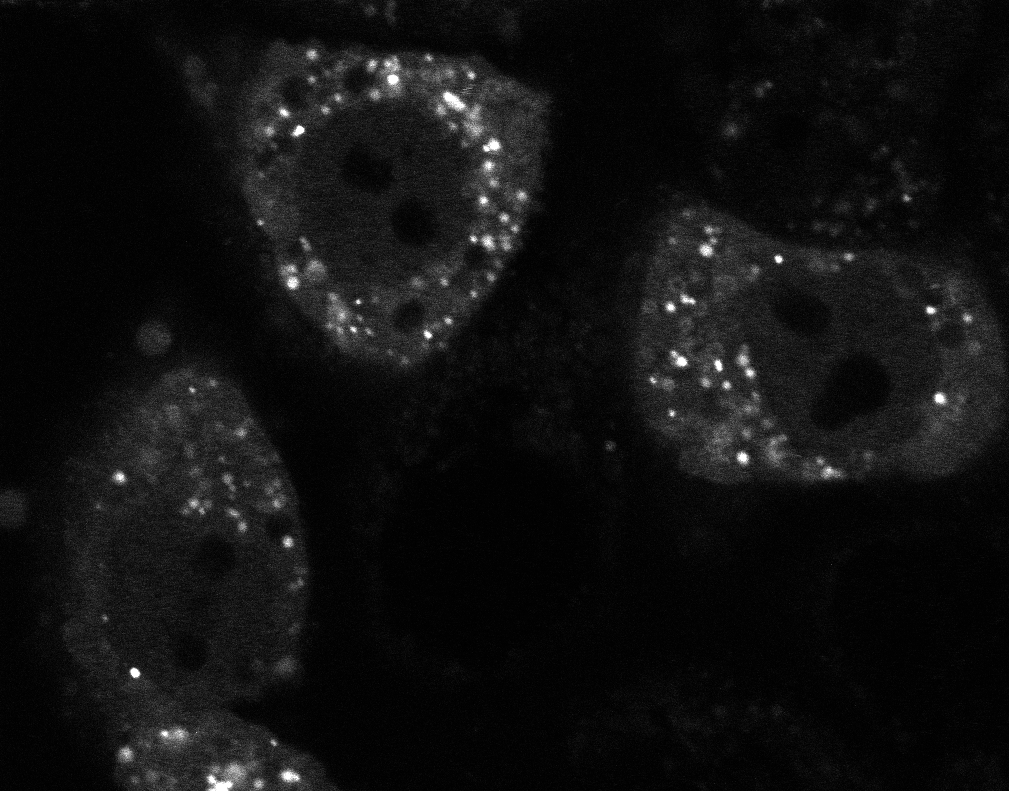

Supplement: Supplementary file 5 — Source data Fig. 2 [file 44318_2024_272_MOESM5_ESM.zip › Figure2/Figure2E/ndp52_hex_2min.tif]

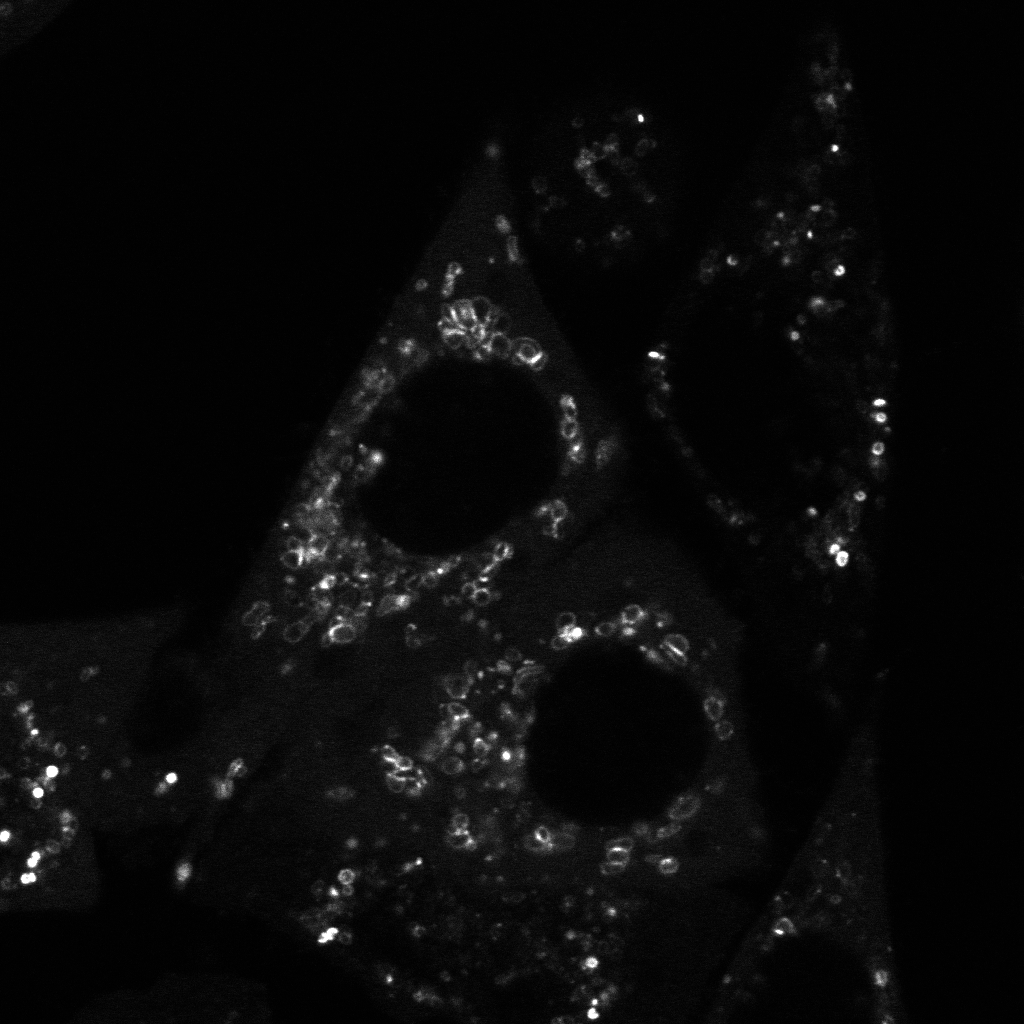

Supplement: Supplementary file 5 — Source data Fig. 2 [file 44318_2024_272_MOESM5_ESM.zip › Figure2/Figure2E/optn.tif]

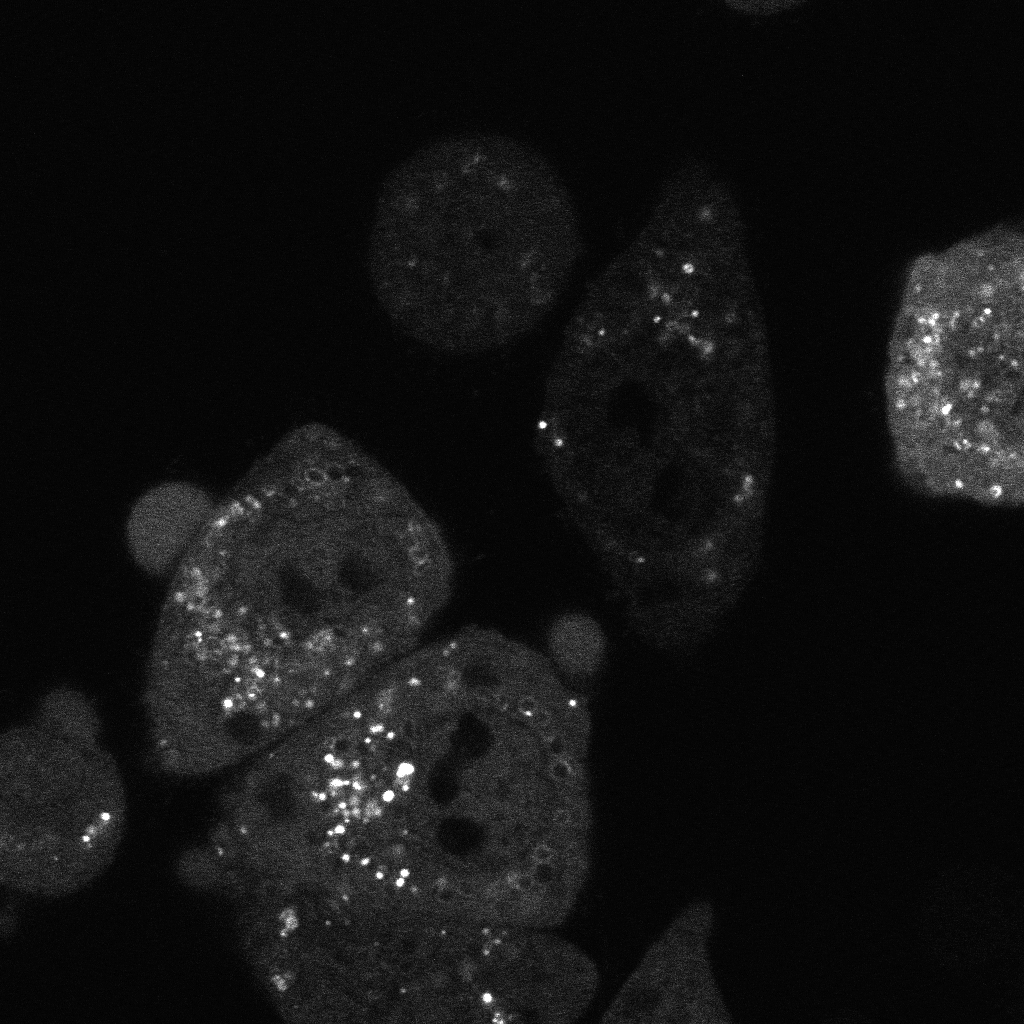

Supplement: Supplementary file 5 — Source data Fig. 2 [file 44318_2024_272_MOESM5_ESM.zip › Figure2/Figure2E/optn_hex_2min.tif]

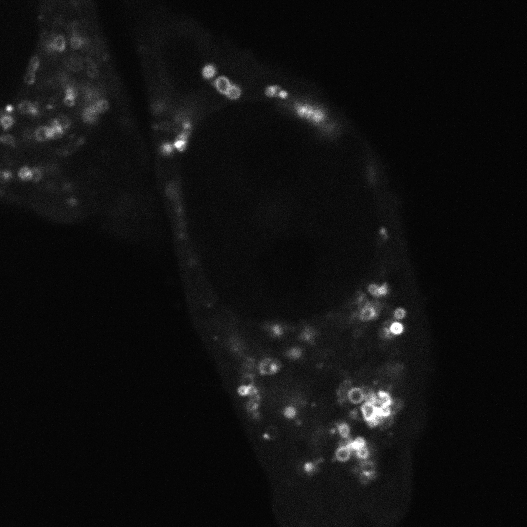

Supplement: Supplementary file 6 — Source data Fig. 4 [file 44318_2024_272_MOESM6_ESM.zip › Figure4/Figure4A/OPTN_same size.tif]

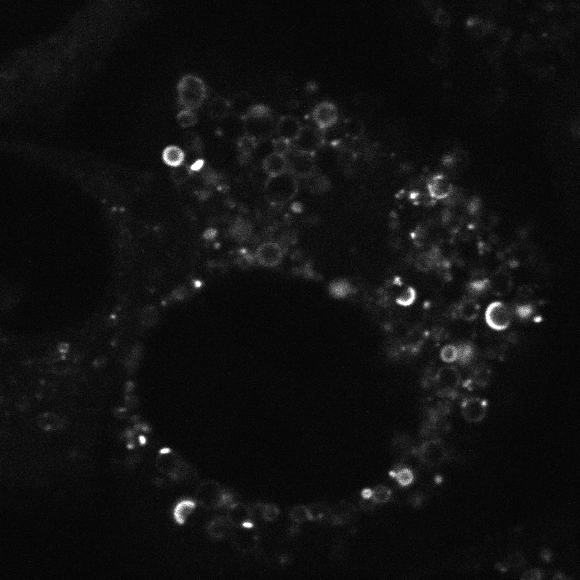

Supplement: Supplementary file 6 — Source data Fig. 4 [file 44318_2024_272_MOESM6_ESM.zip › Figure4/Figure4C/OPTN_different size.tif]

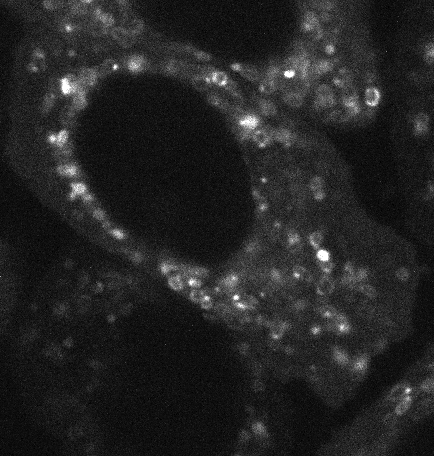

Supplement: Supplementary file 6 — Source data Fig. 4 [file 44318_2024_272_MOESM6_ESM.zip › Figure4/Figure4E/OPTN_Mit-IM.tif]

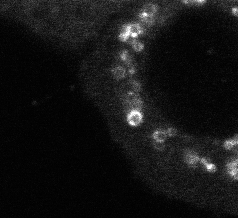

Supplement: Supplementary file 7 — Source data Fig. 5 [file 44318_2024_272_MOESM7_ESM.zip › Figure5/Figure5A/OPTN+nano-Ub.tif]

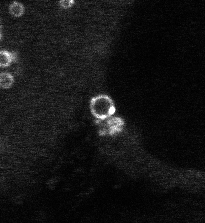

Supplement: Supplementary file 7 — Source data Fig. 5 [file 44318_2024_272_MOESM7_ESM.zip › Figure5/Figure5A/OPTN+Ub.tif]

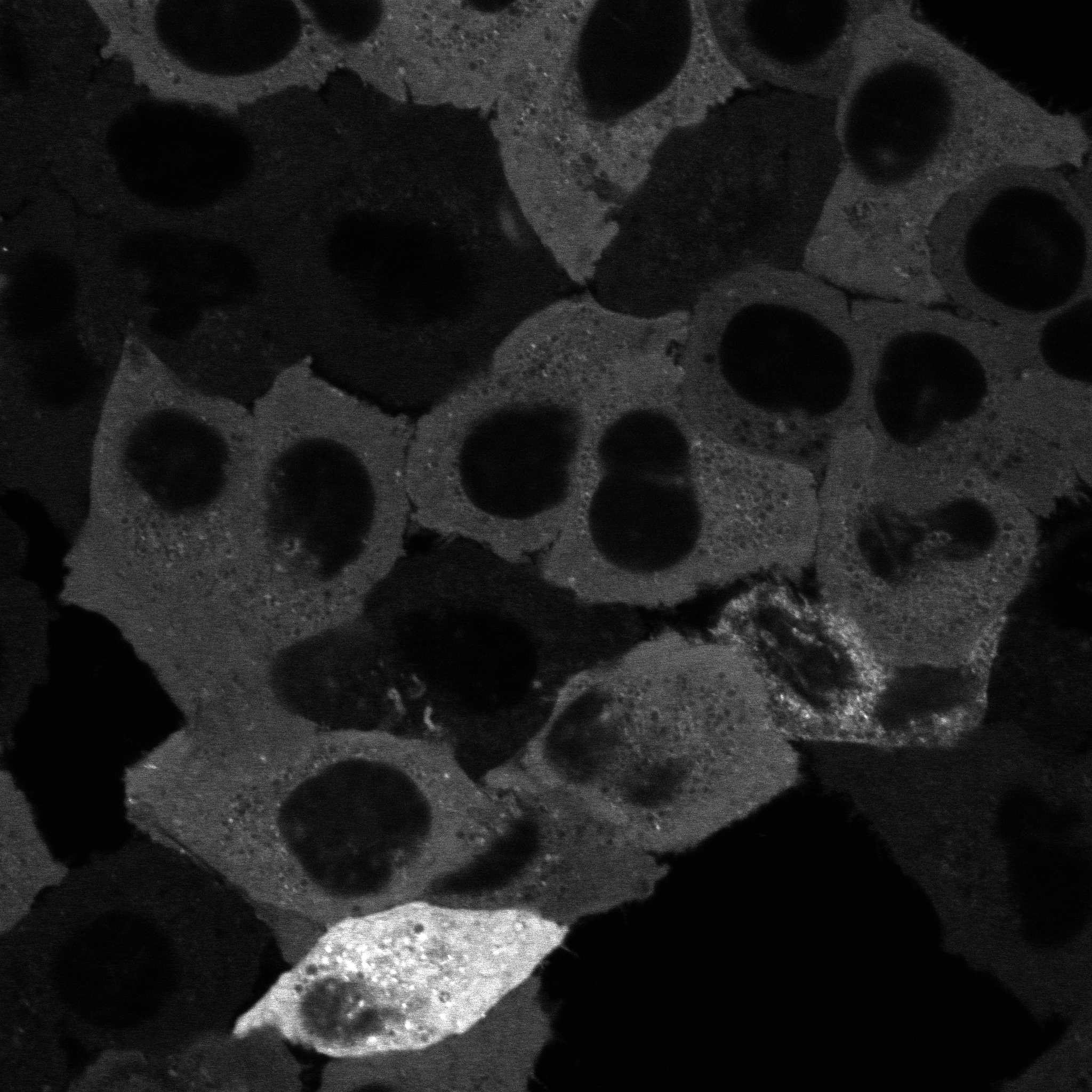

Supplement: Supplementary file 7 — Source data Fig. 5 [file 44318_2024_272_MOESM7_ESM.zip › Figure5/Figure5A/OPTN-uban-del+ub.tif]

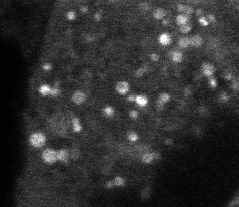

Supplement: Supplementary file 7 — Source data Fig. 5 [file 44318_2024_272_MOESM7_ESM.zip › Figure5/Figure5A/OPTN-udan-del+nanoUb.tif]

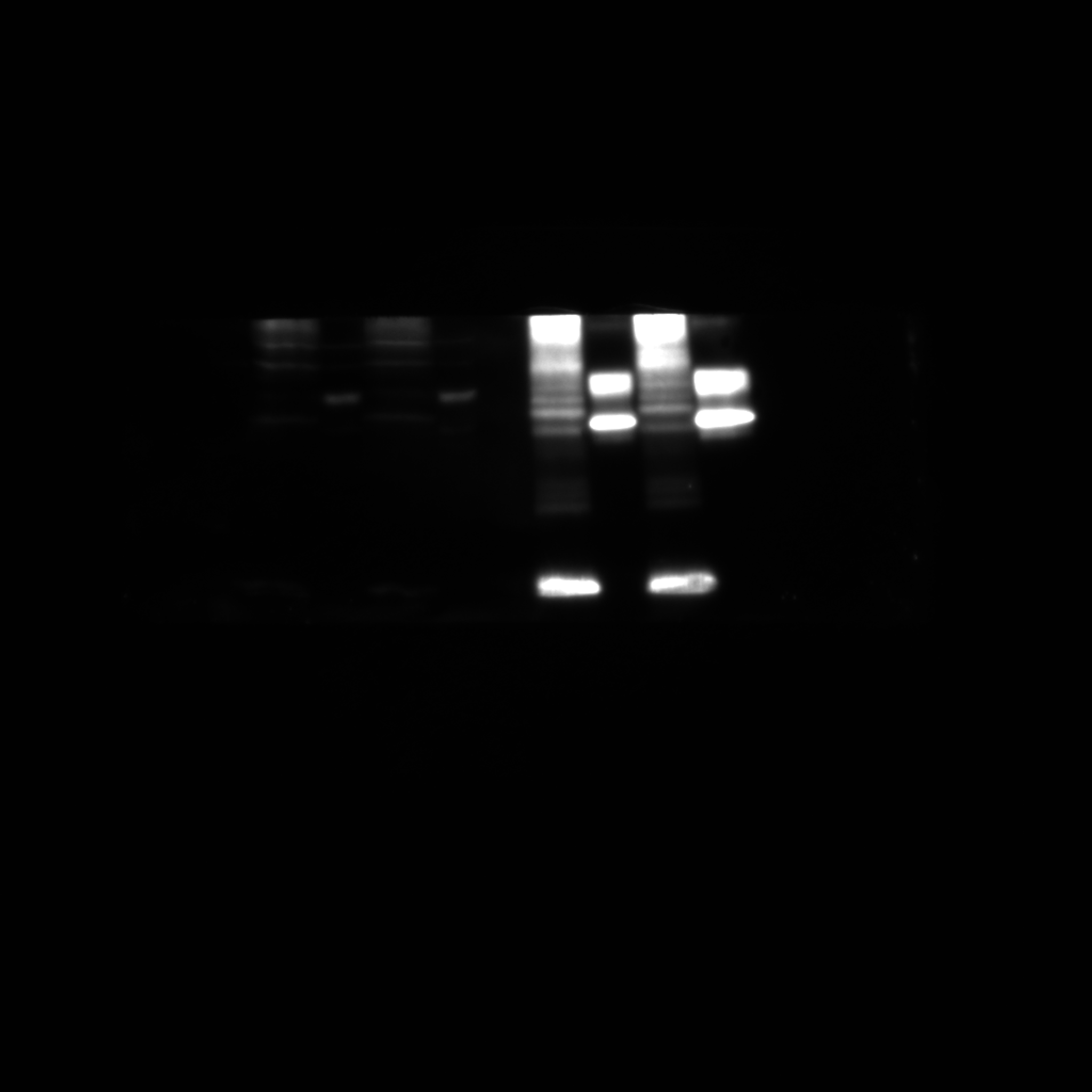

Supplement: Supplementary file 7 — Source data Fig. 5 [file 44318_2024_272_MOESM7_ESM.zip › Figure5/Figure5B/Anti-FLAG-Ub-short exposure.Tif]

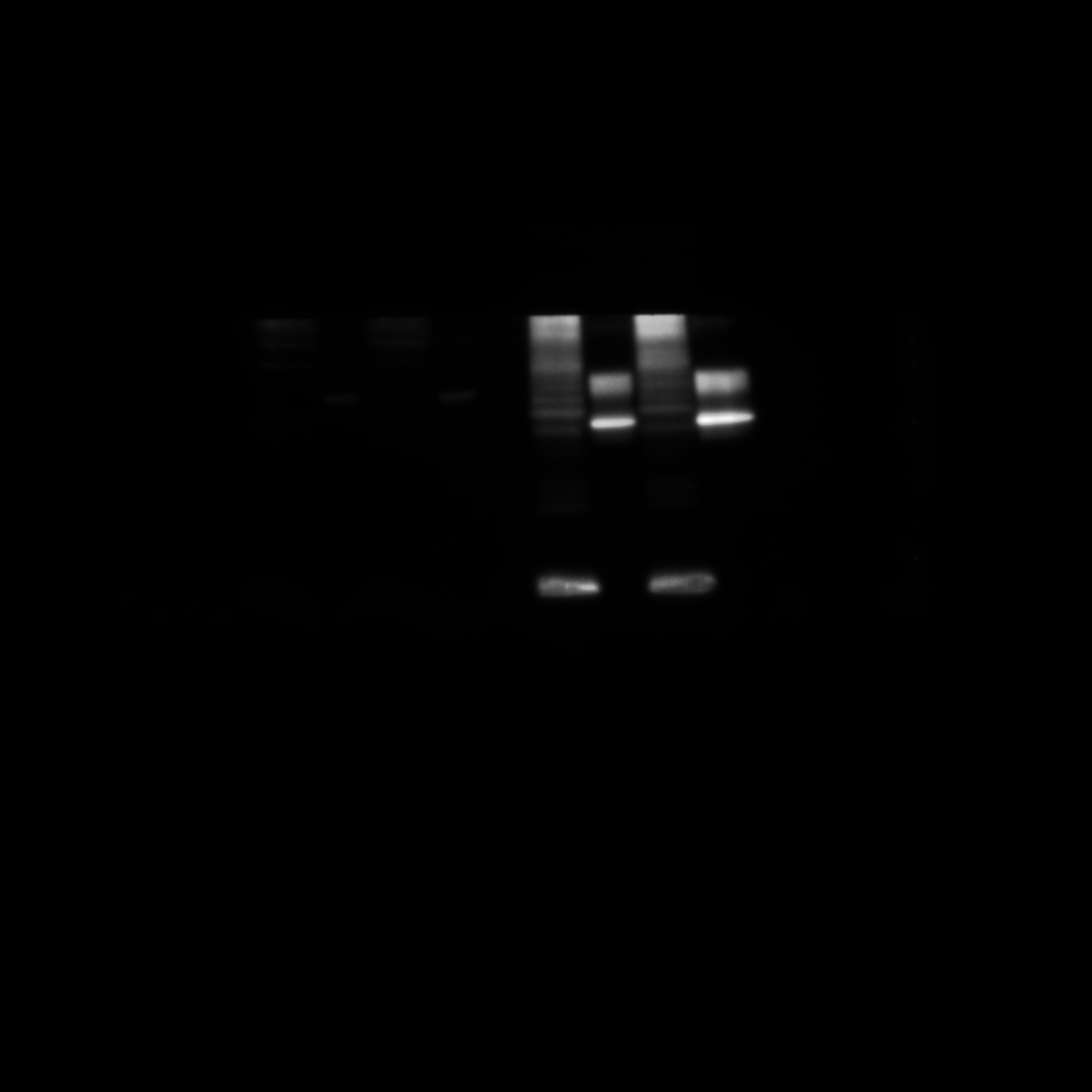

Supplement: Supplementary file 7 — Source data Fig. 5 [file 44318_2024_272_MOESM7_ESM.zip › Figure5/Figure5B/Anti-FLAG-Ub-short expoure-2.Tif]

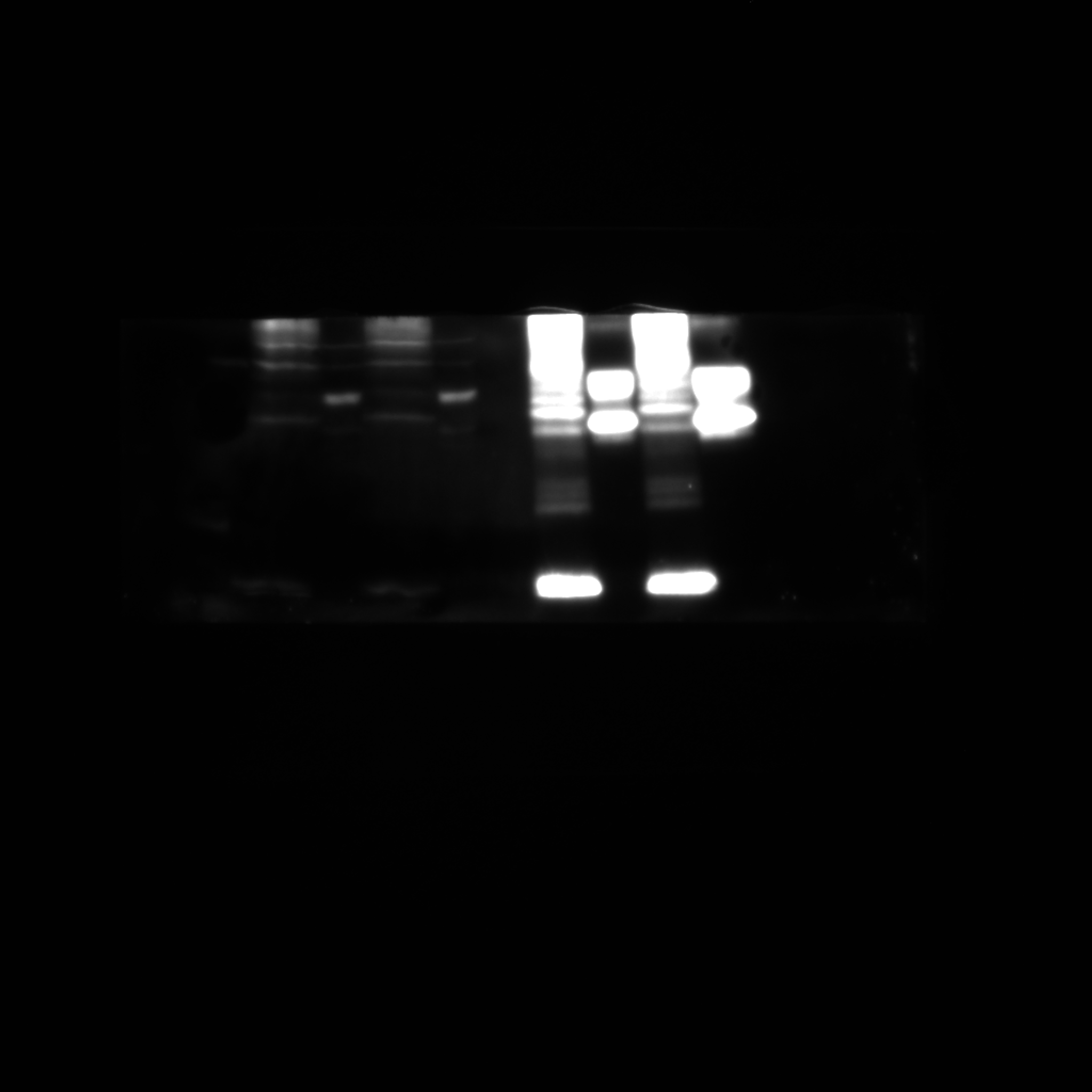

Supplement: Supplementary file 7 — Source data Fig. 5 [file 44318_2024_272_MOESM7_ESM.zip › Figure5/Figure5B/Anti-FLAG-Ub.Tif]

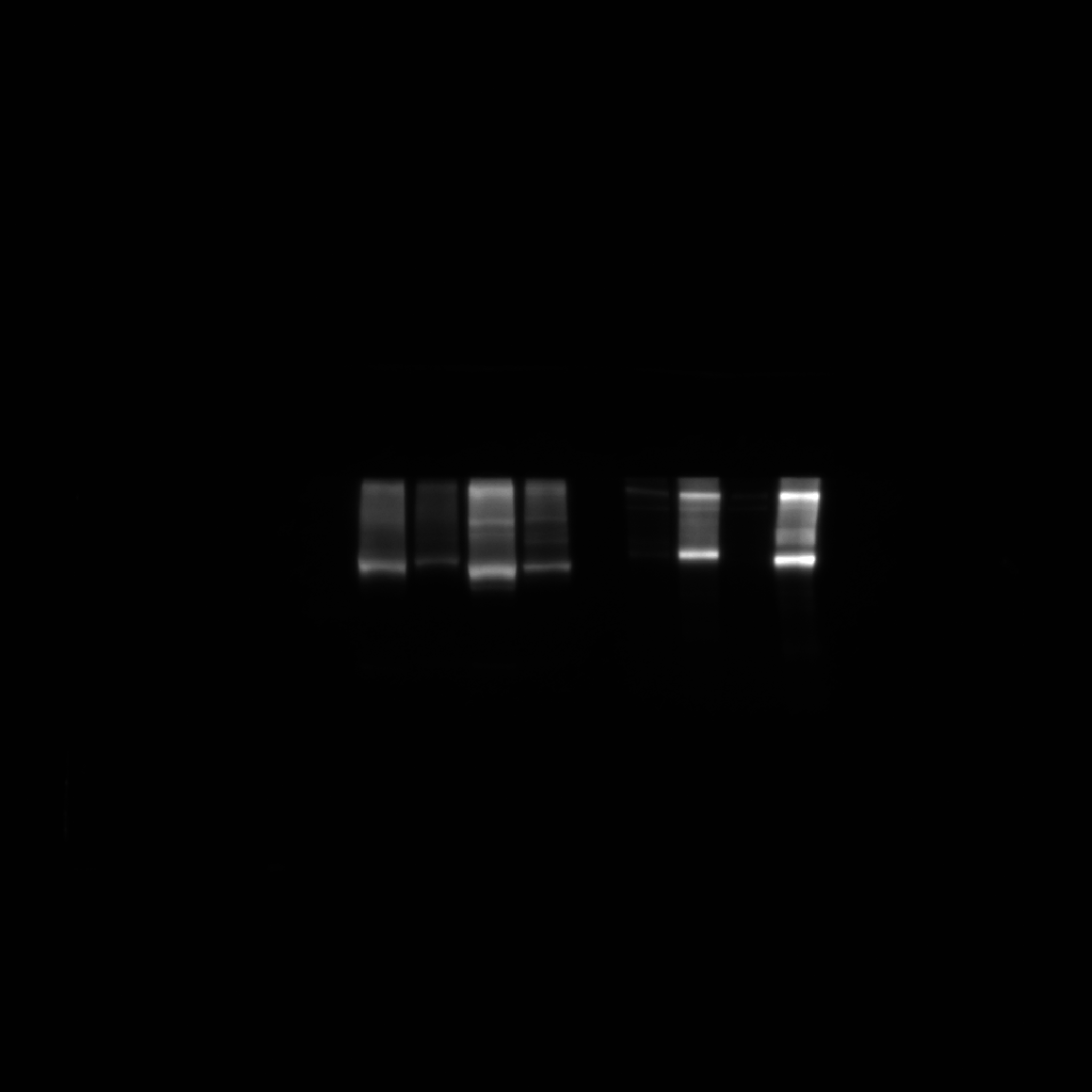

Supplement: Supplementary file 7 — Source data Fig. 5 [file 44318_2024_272_MOESM7_ESM.zip › Figure5/Figure5B/Anti-GFP-optn.Tif]

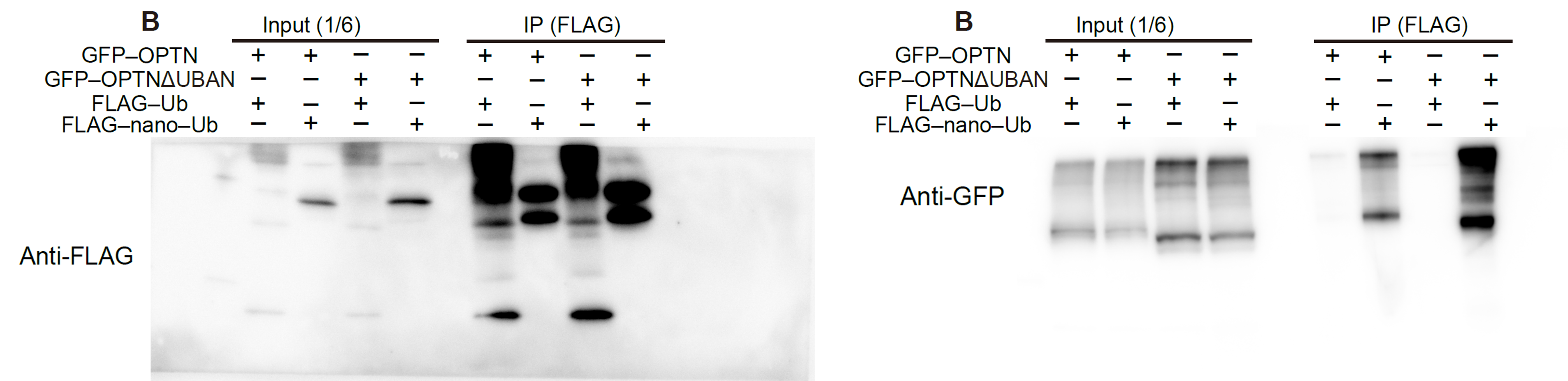

Supplement: Supplementary file 7 — Source data Fig. 5 [file 44318_2024_272_MOESM7_ESM.zip › Figure5/Figure5B/IP .tif]

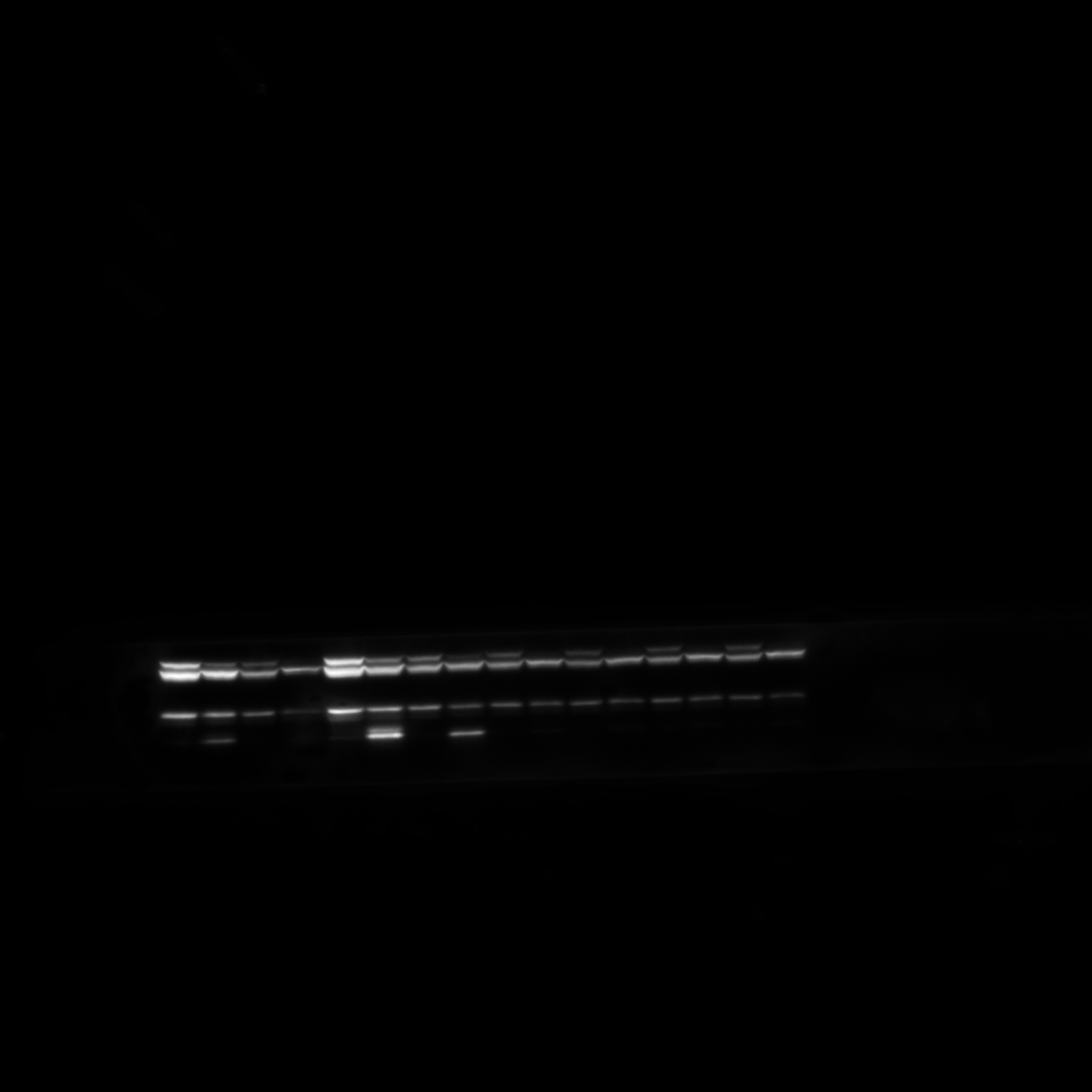

Supplement: Supplementary file 7 — Source data Fig. 5 [file 44318_2024_272_MOESM7_ESM.zip › Figure5/Figure5D/halo.Tif.Tif]

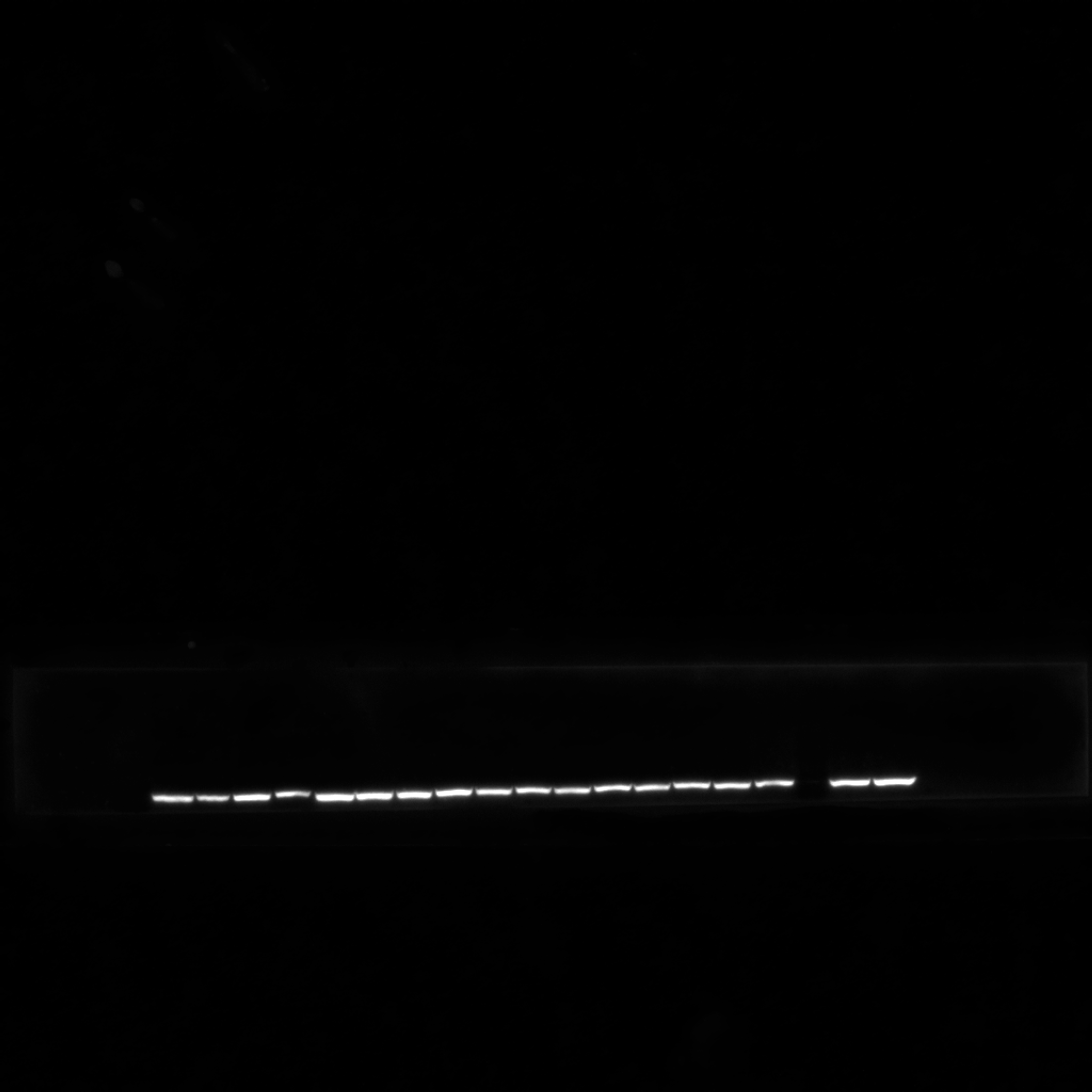

Supplement: Supplementary file 7 — Source data Fig. 5 [file 44318_2024_272_MOESM7_ESM.zip › Figure5/Figure5D/hsp.Tif]

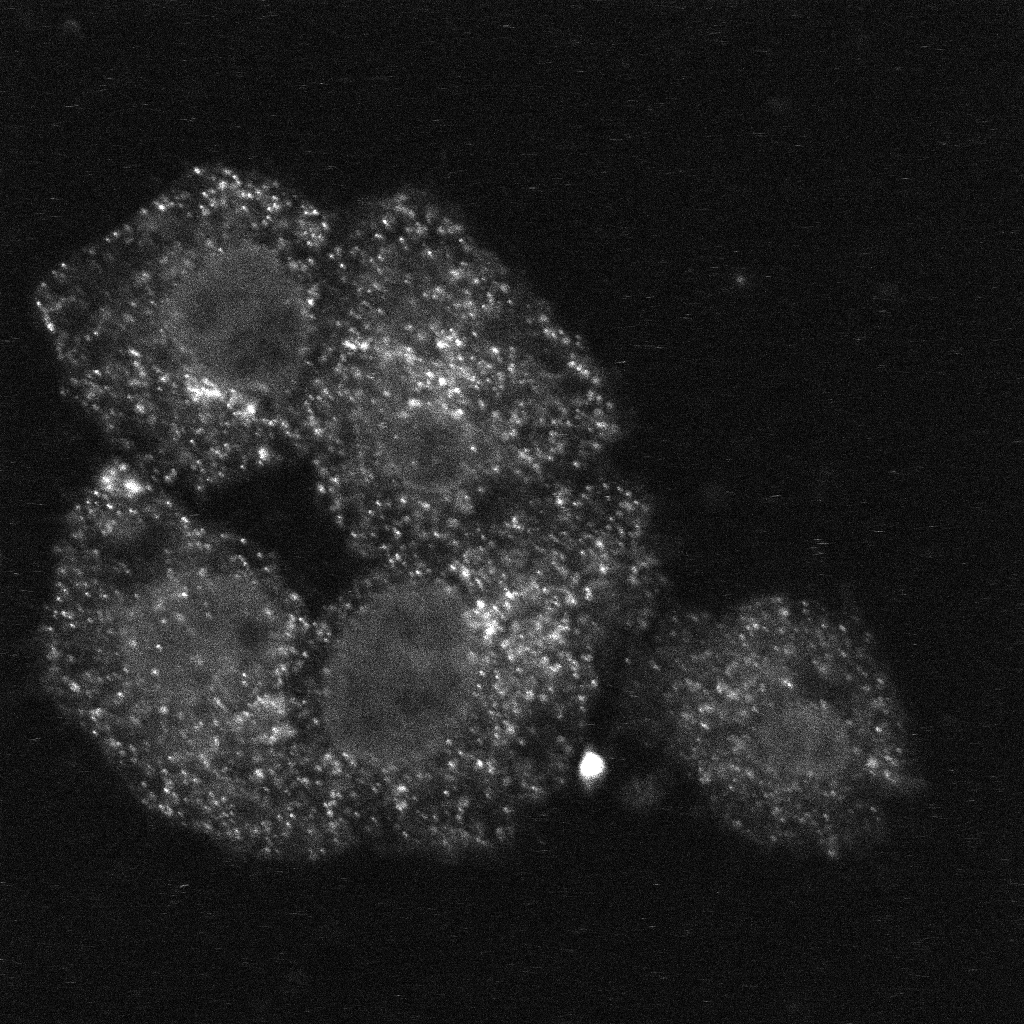

Supplement: Supplementary file 8 — Source data Fig. 6 [file 44318_2024_272_MOESM8_ESM.zip › Figure6/Figure6A/With CCCP/OPTN-uban-del+nano-Ub+CCCP.tif]

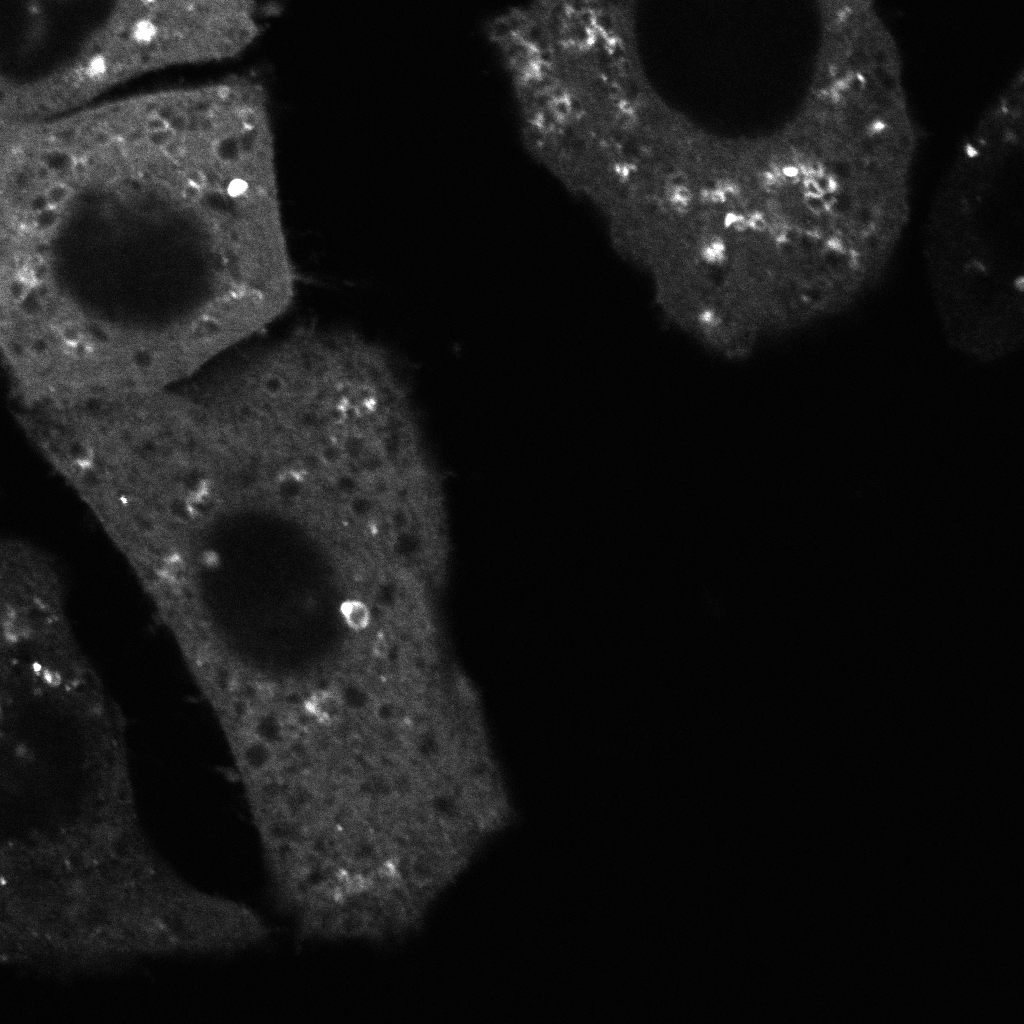

Supplement: Supplementary file 8 — Source data Fig. 6 [file 44318_2024_272_MOESM8_ESM.zip › Figure6/Figure6A/With CCCP/optn+ub+cccp_0010.tif]

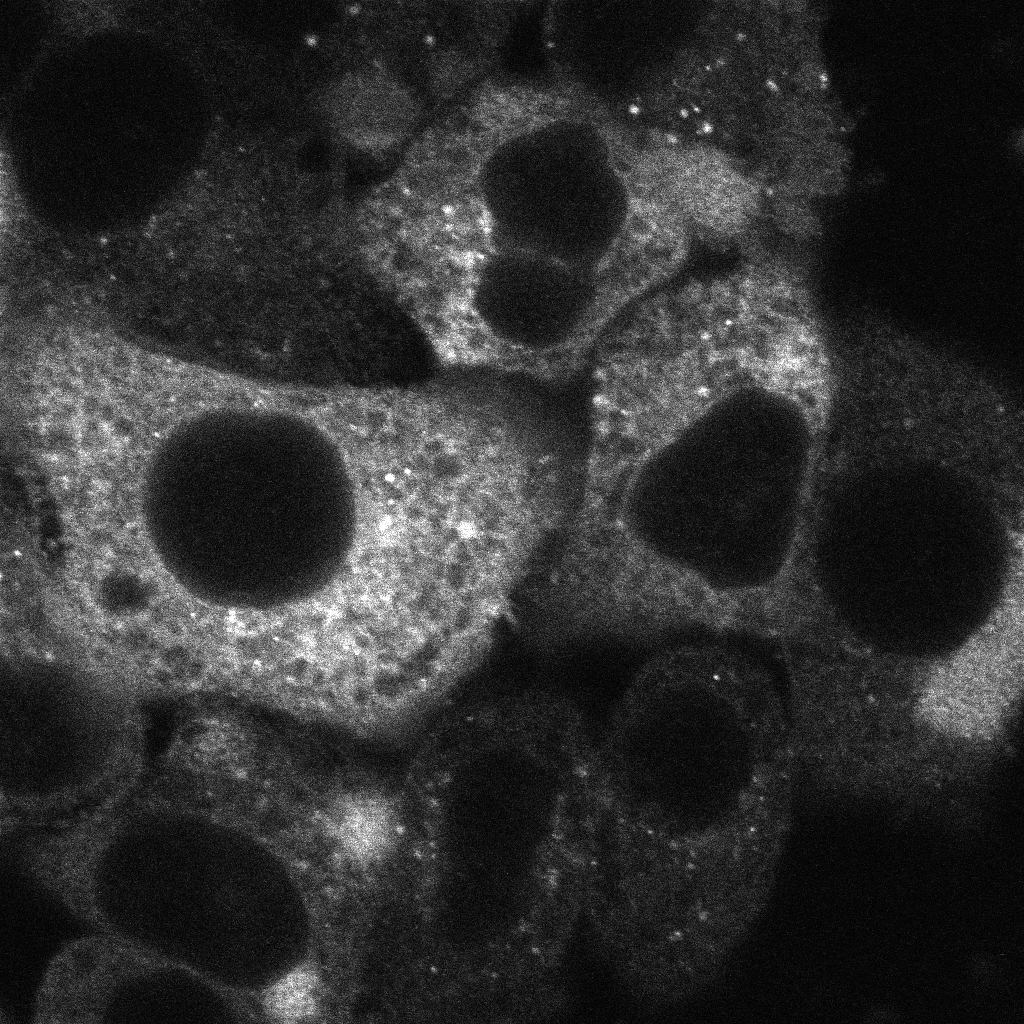

Supplement: Supplementary file 8 — Source data Fig. 6 [file 44318_2024_272_MOESM8_ESM.zip › Figure6/Figure6A/Without CCCP/optn+ub-No cccp.tif]

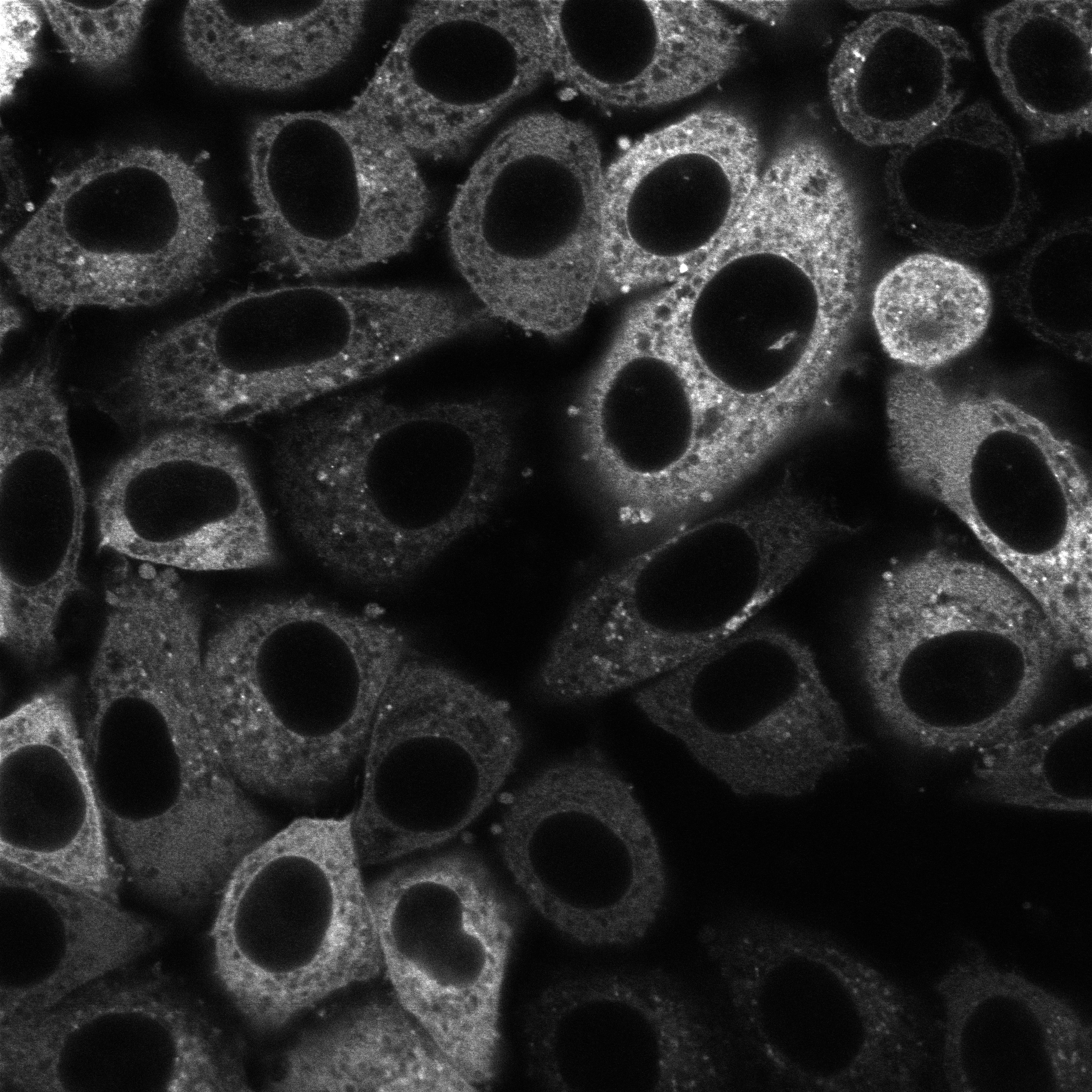

Supplement: Supplementary file 8 — Source data Fig. 6 [file 44318_2024_272_MOESM8_ESM.zip › Figure6/Figure6A/Without CCCP/optn-uban-del+nanoub-No cccp.tif]

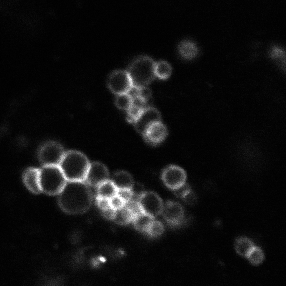

Supplement: Supplementary file 9 — EV Figure Source Data [file 44318_2024_272_MOESM9_ESM.zip › SourceData_allEV/FigureEV1/A/NBR1.tif]

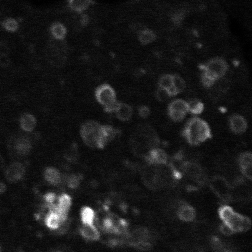

Supplement: Supplementary file 9 — EV Figure Source Data [file 44318_2024_272_MOESM9_ESM.zip › SourceData_allEV/FigureEV1/A/NDP52.tif]

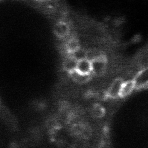

Supplement: Supplementary file 9 — EV Figure Source Data [file 44318_2024_272_MOESM9_ESM.zip › SourceData_allEV/FigureEV1/A/OPTN.tif]

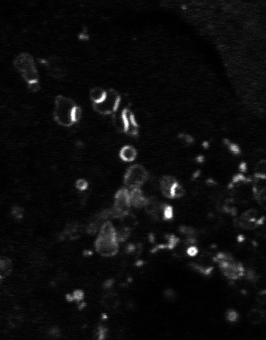

Supplement: Supplementary file 9 — EV Figure Source Data [file 44318_2024_272_MOESM9_ESM.zip › SourceData_allEV/FigureEV1/A/p62.tif]

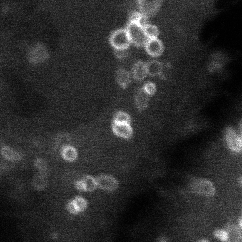

Supplement: Supplementary file 9 — EV Figure Source Data [file 44318_2024_272_MOESM9_ESM.zip › SourceData_allEV/FigureEV1/A/TAX1BP1.tif]

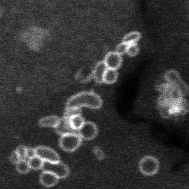

Supplement: Supplementary file 9 — EV Figure Source Data [file 44318_2024_272_MOESM9_ESM.zip › SourceData_allEV/FigureEV1/A/ub.tif]

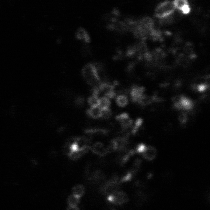

Supplement: Supplementary file 9 — EV Figure Source Data [file 44318_2024_272_MOESM9_ESM.zip › SourceData_allEV/FigureEV1/C/NBR1.tif]

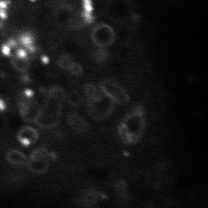

Supplement: Supplementary file 9 — EV Figure Source Data [file 44318_2024_272_MOESM9_ESM.zip › SourceData_allEV/FigureEV1/C/NDP52.tif]

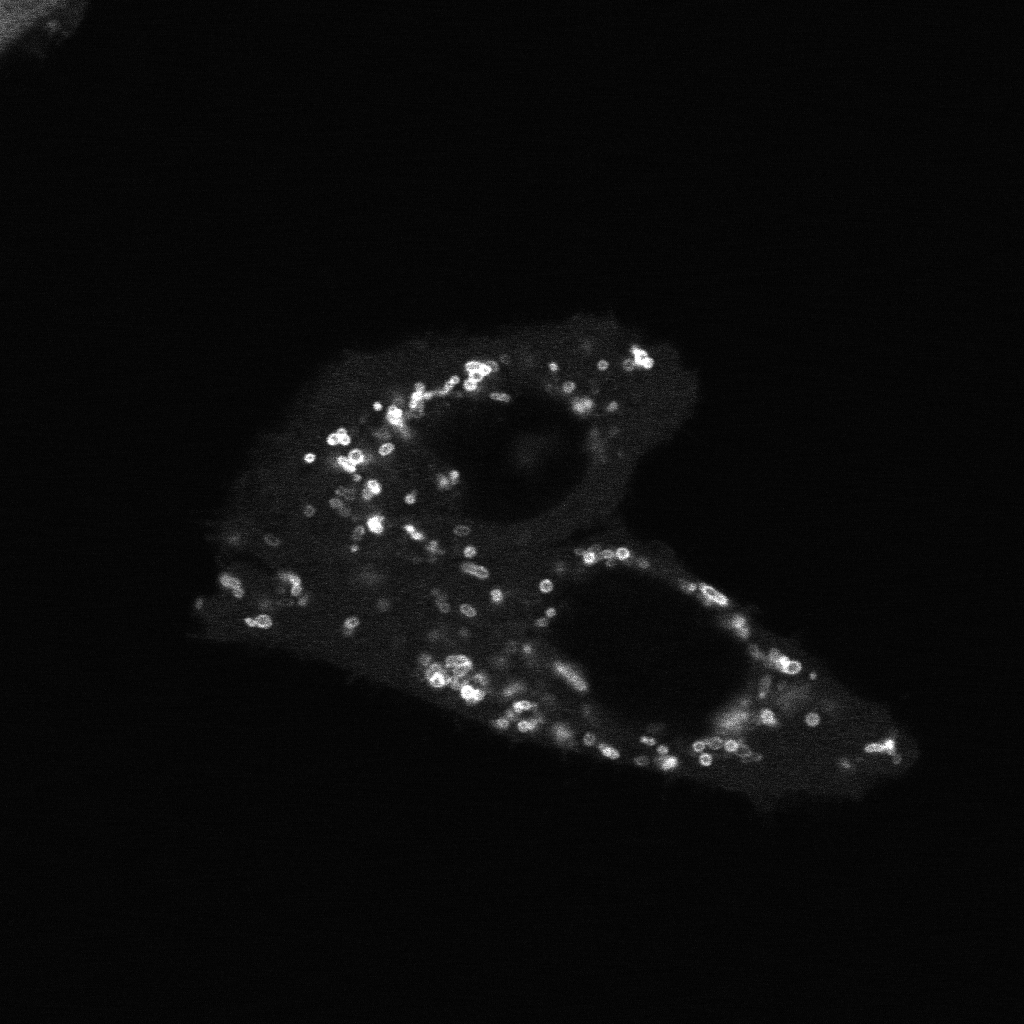

Supplement: Supplementary file 9 — EV Figure Source Data [file 44318_2024_272_MOESM9_ESM.zip › SourceData_allEV/FigureEV1/C/OPTN.tif]

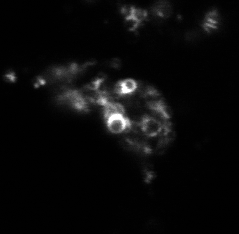

Supplement: Supplementary file 9 — EV Figure Source Data [file 44318_2024_272_MOESM9_ESM.zip › SourceData_allEV/FigureEV1/C/p62.tif]

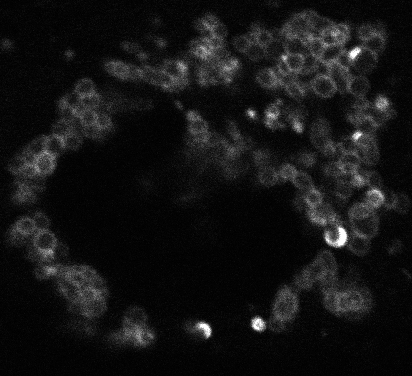

Supplement: Supplementary file 9 — EV Figure Source Data [file 44318_2024_272_MOESM9_ESM.zip › SourceData_allEV/FigureEV1/C/TAX1BP1.tif]

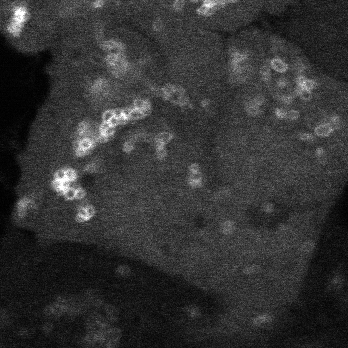

Supplement: Supplementary file 9 — EV Figure Source Data [file 44318_2024_272_MOESM9_ESM.zip › SourceData_allEV/FigureEV1/C/ub.tif]

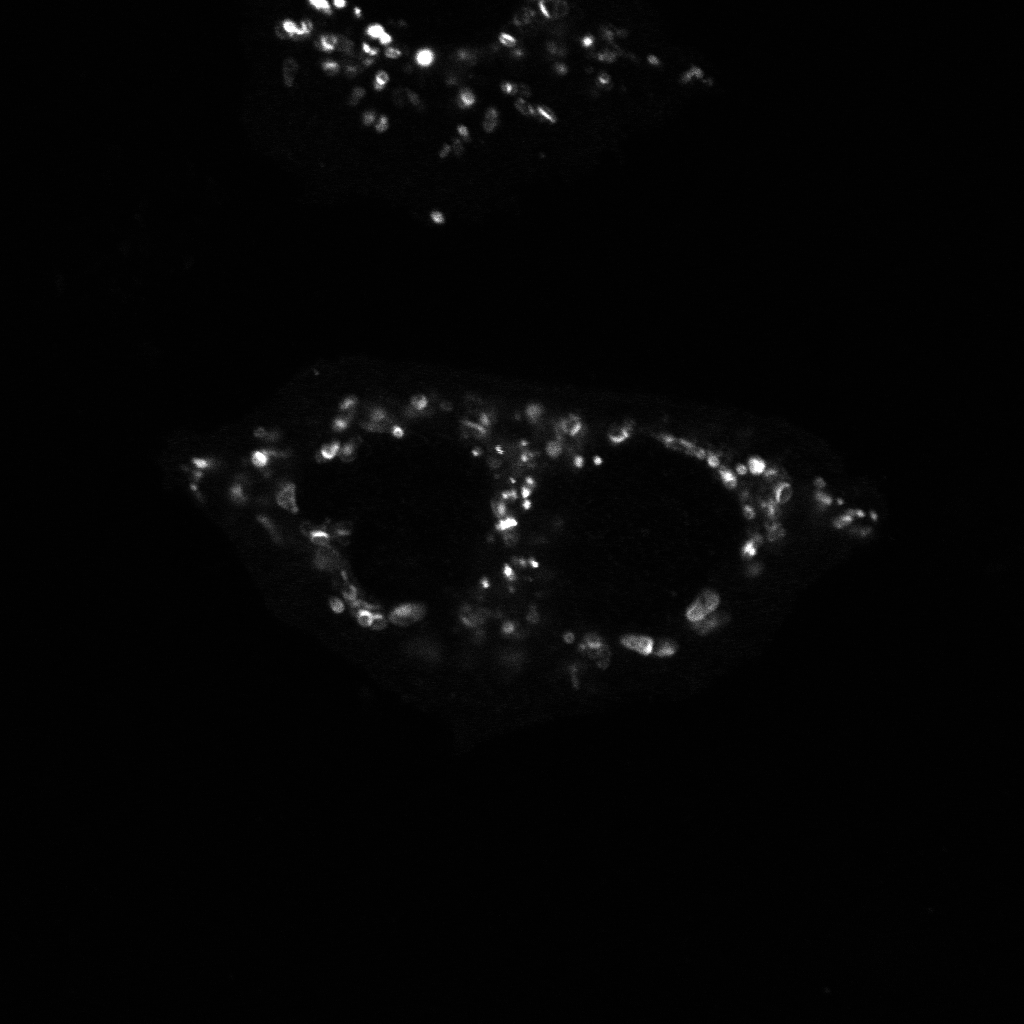

Supplement: Supplementary file 9 — EV Figure Source Data [file 44318_2024_272_MOESM9_ESM.zip › SourceData_allEV/FigureEV2/A/p62-cccp-hex0min.tif]

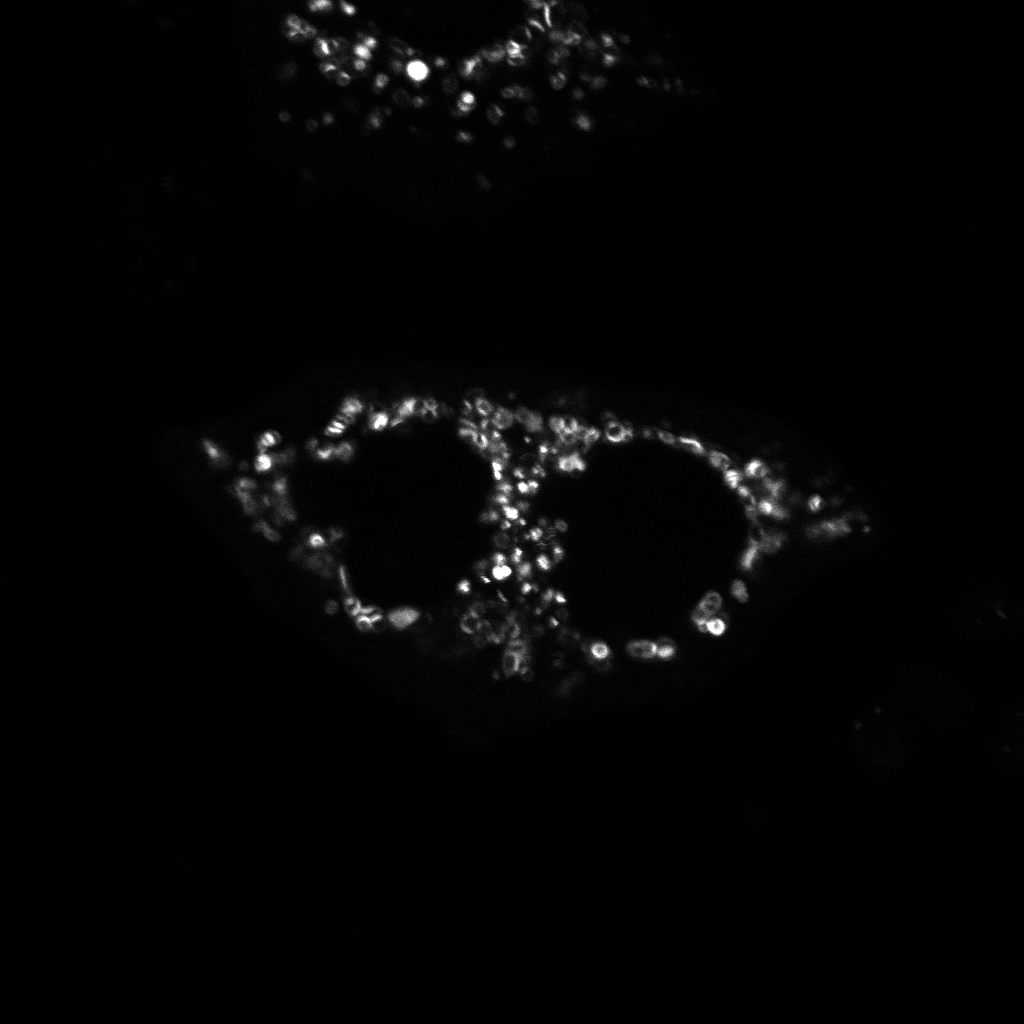

Supplement: Supplementary file 9 — EV Figure Source Data [file 44318_2024_272_MOESM9_ESM.zip › SourceData_allEV/FigureEV2/A/p62-cccp-hex2min.tif]

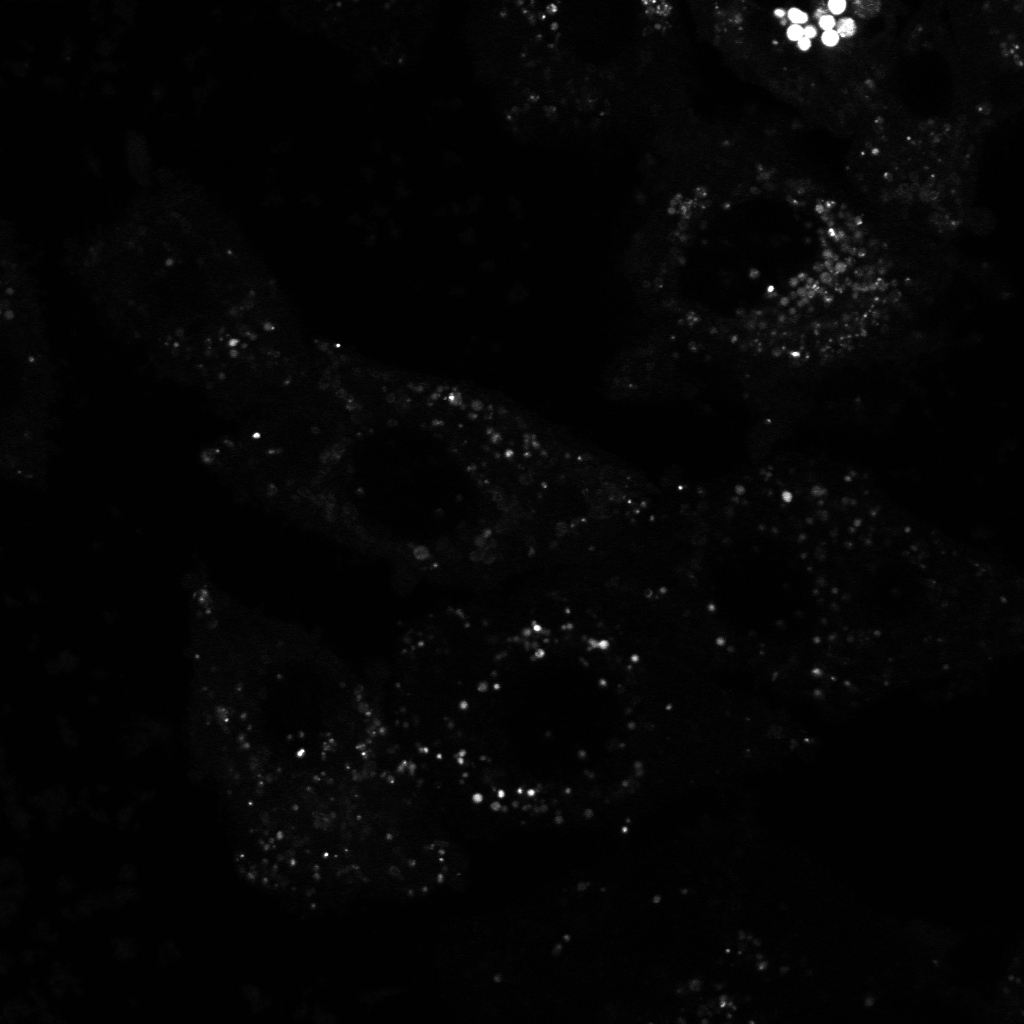

Supplement: Supplementary file 9 — EV Figure Source Data [file 44318_2024_272_MOESM9_ESM.zip › SourceData_allEV/FigureEV2/B/ndp52-Hex0min.tif]

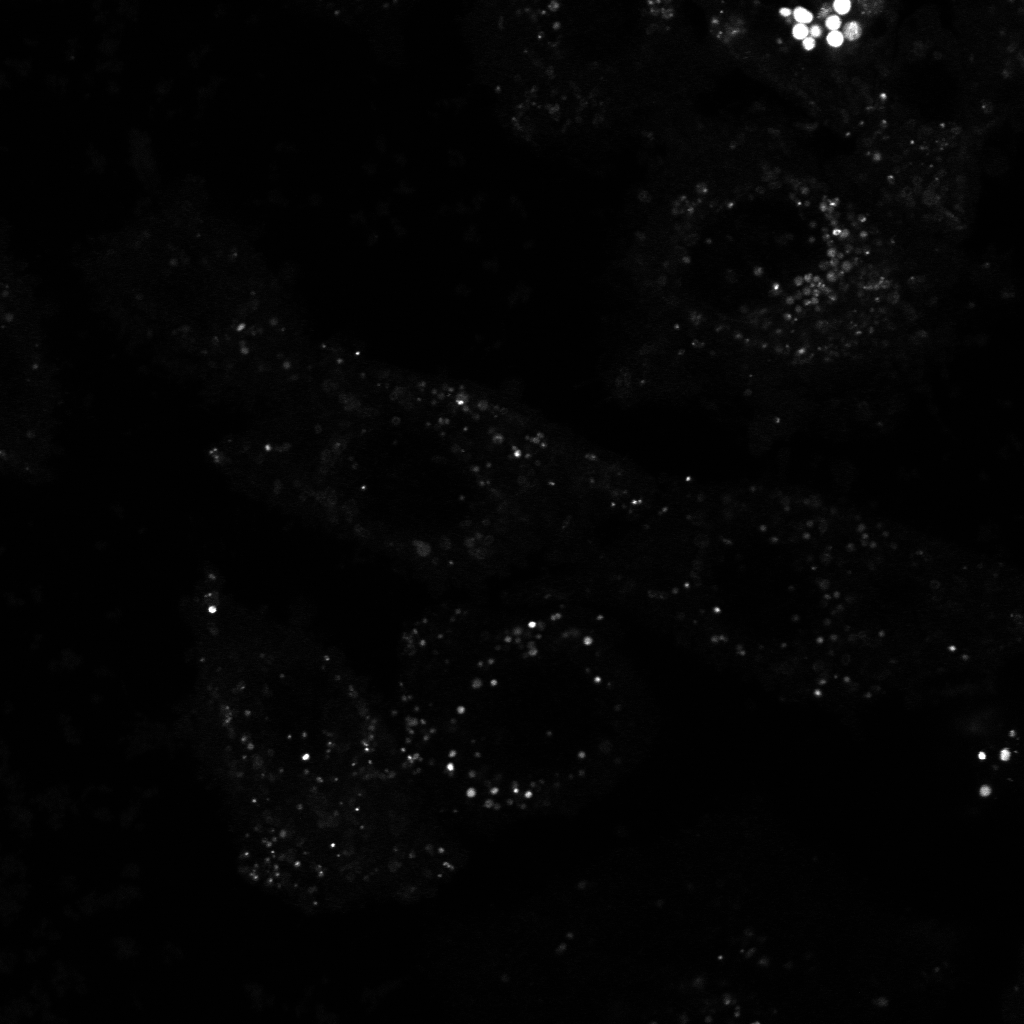

Supplement: Supplementary file 9 — EV Figure Source Data [file 44318_2024_272_MOESM9_ESM.zip › SourceData_allEV/FigureEV2/B/ndp52-Hex2min.tif]

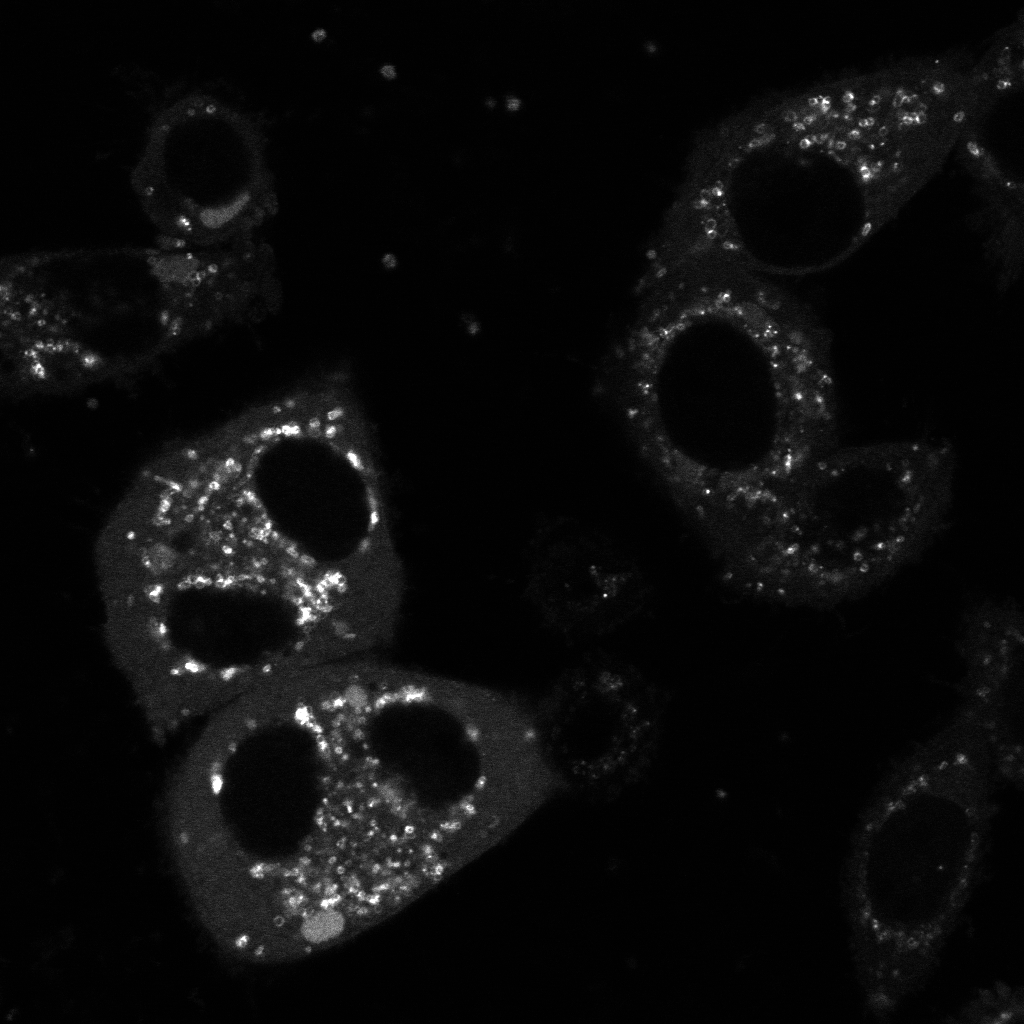

Supplement: Supplementary file 9 — EV Figure Source Data [file 44318_2024_272_MOESM9_ESM.zip › SourceData_allEV/FigureEV2/B/optn-Hex0min.tif]

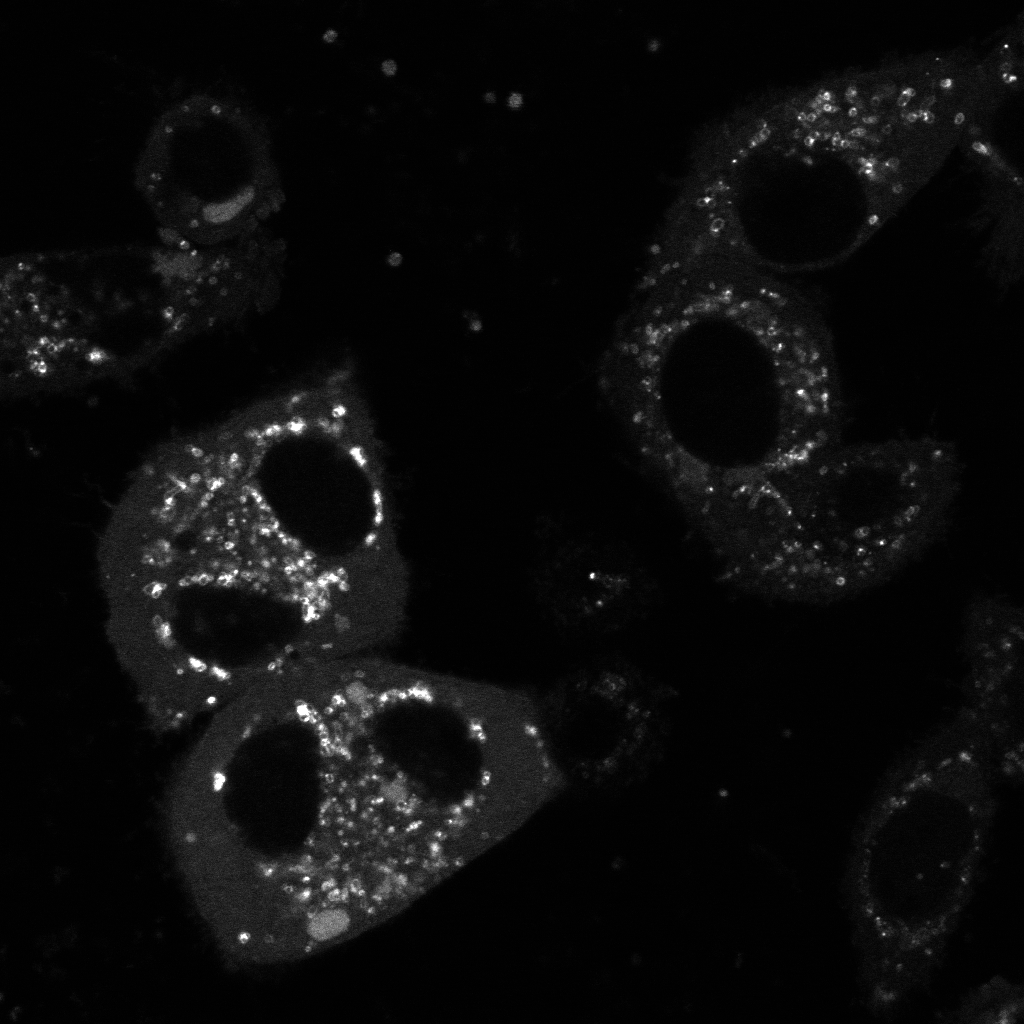

Supplement: Supplementary file 9 — EV Figure Source Data [file 44318_2024_272_MOESM9_ESM.zip › SourceData_allEV/FigureEV2/B/optn-Hex2min.tif]

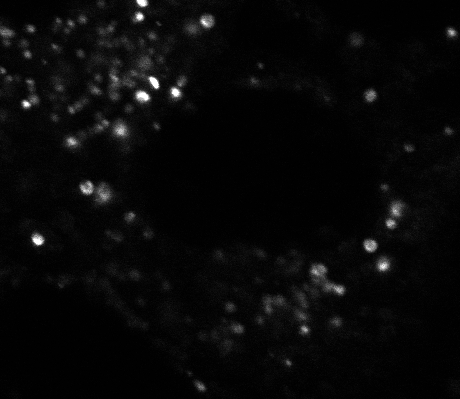

Supplement: Supplementary file 9 — EV Figure Source Data [file 44318_2024_272_MOESM9_ESM.zip › SourceData_allEV/FigureEV2/C/NDP52-Hex0min.tif]

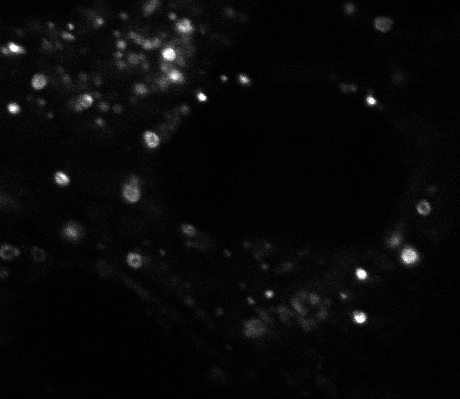

Supplement: Supplementary file 9 — EV Figure Source Data [file 44318_2024_272_MOESM9_ESM.zip › SourceData_allEV/FigureEV2/C/NDP52-Hex2min.tif]

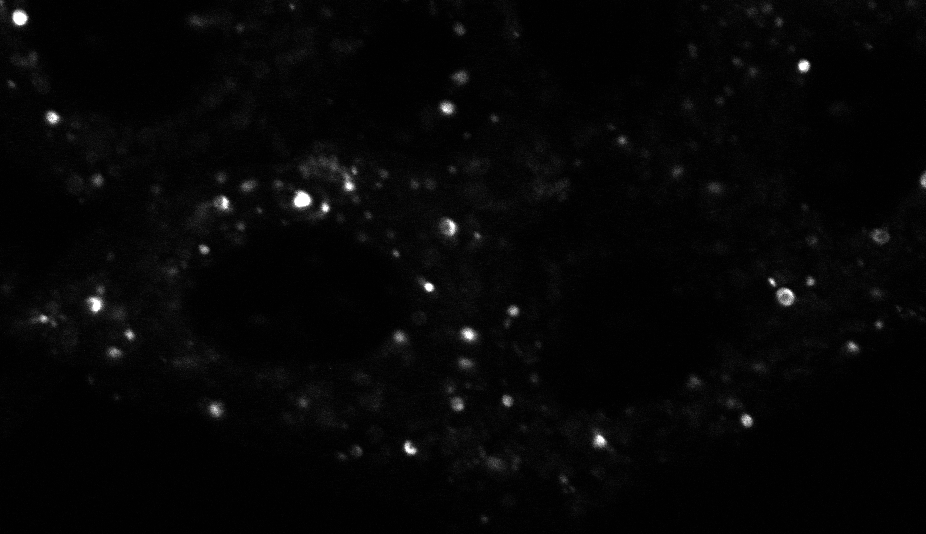

Supplement: Supplementary file 9 — EV Figure Source Data [file 44318_2024_272_MOESM9_ESM.zip › SourceData_allEV/FigureEV2/C/OPTN-Hex0min.tif]

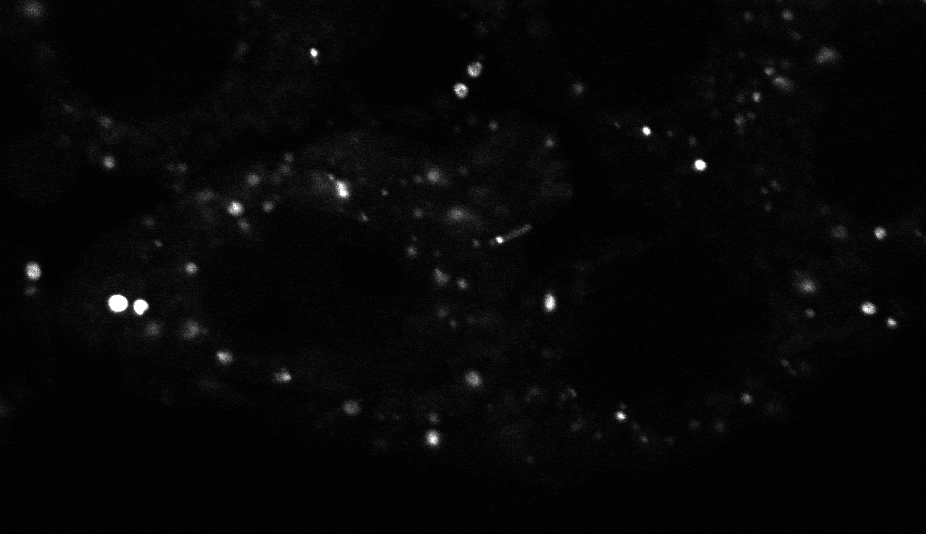

Supplement: Supplementary file 9 — EV Figure Source Data [file 44318_2024_272_MOESM9_ESM.zip › SourceData_allEV/FigureEV2/C/OPTN-Hex2min.tif]

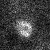

Supplement: Supplementary file 9 — EV Figure Source Data [file 44318_2024_272_MOESM9_ESM.zip › SourceData_allEV/FigureEV4/A/GFP-OPTN-dUBD-Nano-Ub/20240726-GFP-OPTN-dUBD-Nano-Ub_0010_LSM-bleaching.tif]

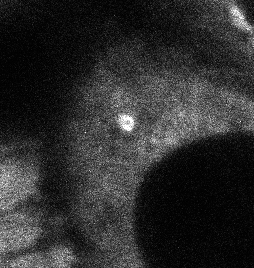

Supplement: Supplementary file 9 — EV Figure Source Data [file 44318_2024_272_MOESM9_ESM.zip › SourceData_allEV/FigureEV4/A/GFP-OPTN-dUBD-Nano-Ub/20240726-GFP-OPTN-dUBD-Nano-Ub_0010_LSM.tif]

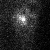

Supplement: Supplementary file 9 — EV Figure Source Data [file 44318_2024_272_MOESM9_ESM.zip › SourceData_allEV/FigureEV4/A/OPTN-dUBD-GFP-Nano-Ub/20240726-OPTN-dUBD-GFP-Nano-Ub_0004_LSM-bleaching.tif]

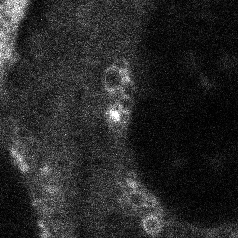

Supplement: Supplementary file 9 — EV Figure Source Data [file 44318_2024_272_MOESM9_ESM.zip › SourceData_allEV/FigureEV4/A/OPTN-dUBD-GFP-Nano-Ub/20240726-OPTN-dUBD-GFP-Nano-Ub_0004_LSM.tif]

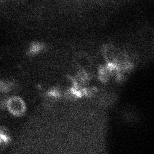

Supplement: Supplementary file 9 — EV Figure Source Data [file 44318_2024_272_MOESM9_ESM.zip › SourceData_allEV/FigureEV4/A/OPTN-Ub/20240726-WT OPTN_Ub_0007_LSM-1.tif]

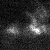

Supplement: Supplementary file 9 — EV Figure Source Data [file 44318_2024_272_MOESM9_ESM.zip › SourceData_allEV/FigureEV4/A/OPTN-Ub/20240726-WT OPTN_Ub_0007_LSM-bleaching.tif]

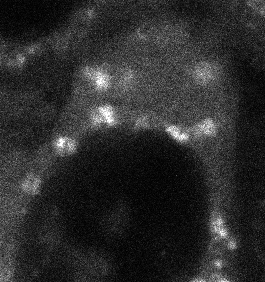

Supplement: Supplementary file 9 — EV Figure Source Data [file 44318_2024_272_MOESM9_ESM.zip › SourceData_allEV/FigureEV4/A/OPTNE478G+Nano-Ub.tif]

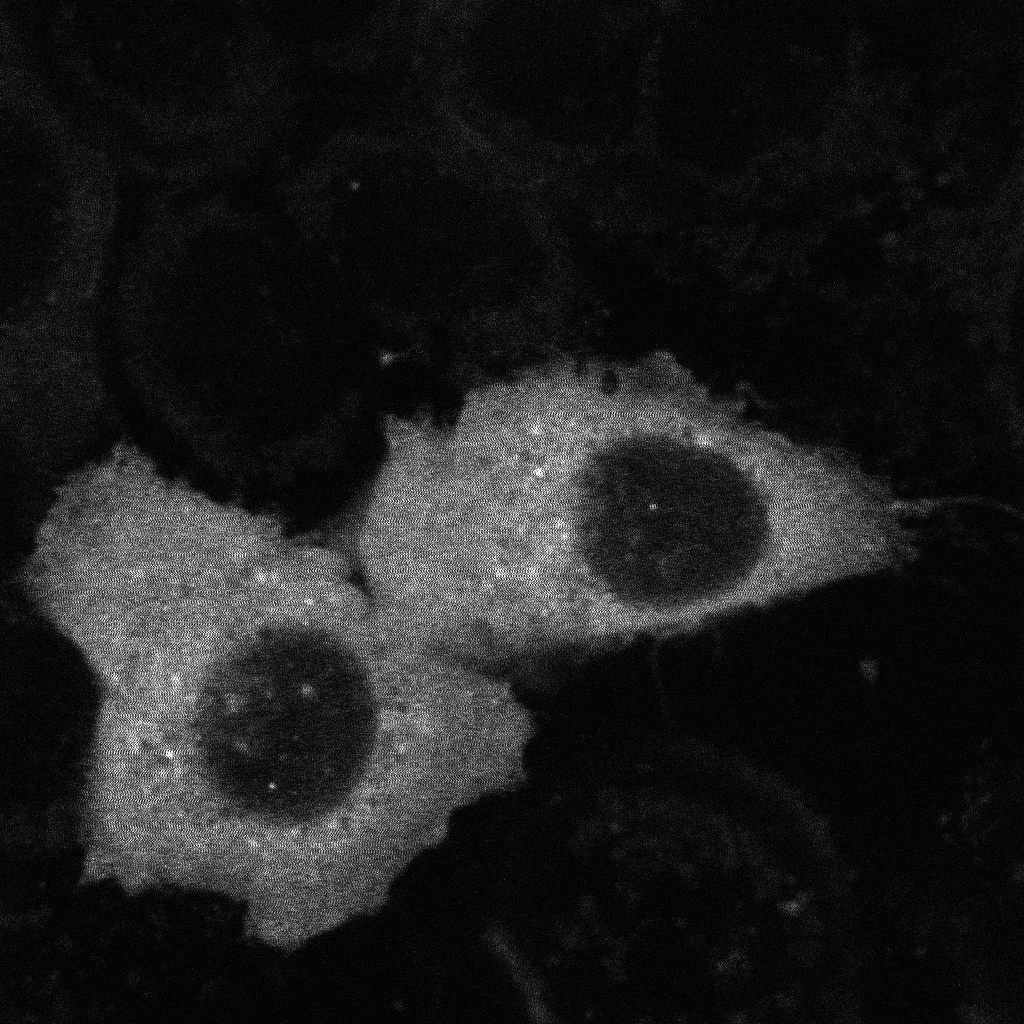

Supplement: Supplementary file 9 — EV Figure Source Data [file 44318_2024_272_MOESM9_ESM.zip › SourceData_allEV/FigureEV4/A/OPTNE478G+Ub.tif]

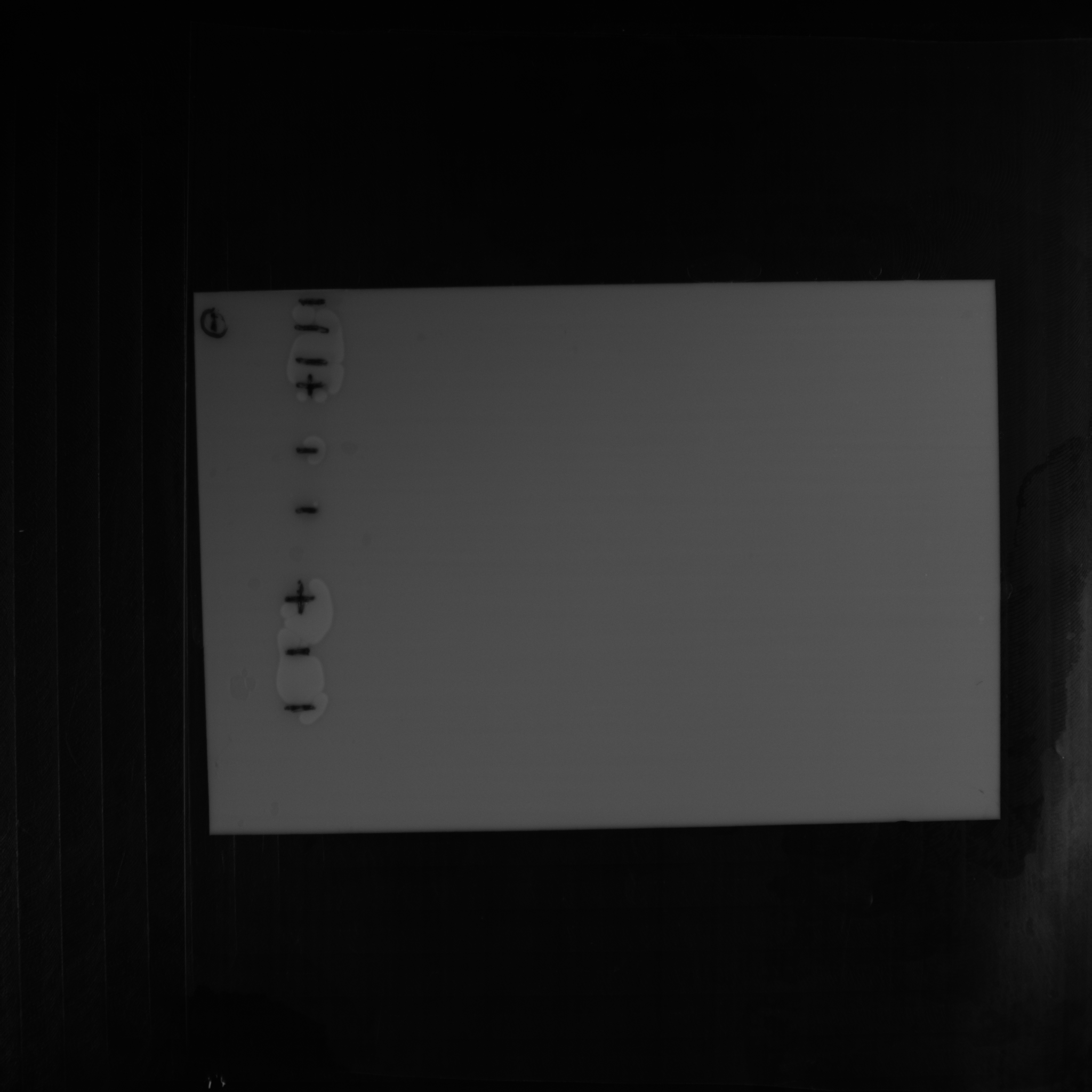

Supplement: Supplementary file 9 — EV Figure Source Data [file 44318_2024_272_MOESM9_ESM.zip › SourceData_allEV/FigureEV4/C/20240722-mitophagy flux/1-actin-M.Tif]

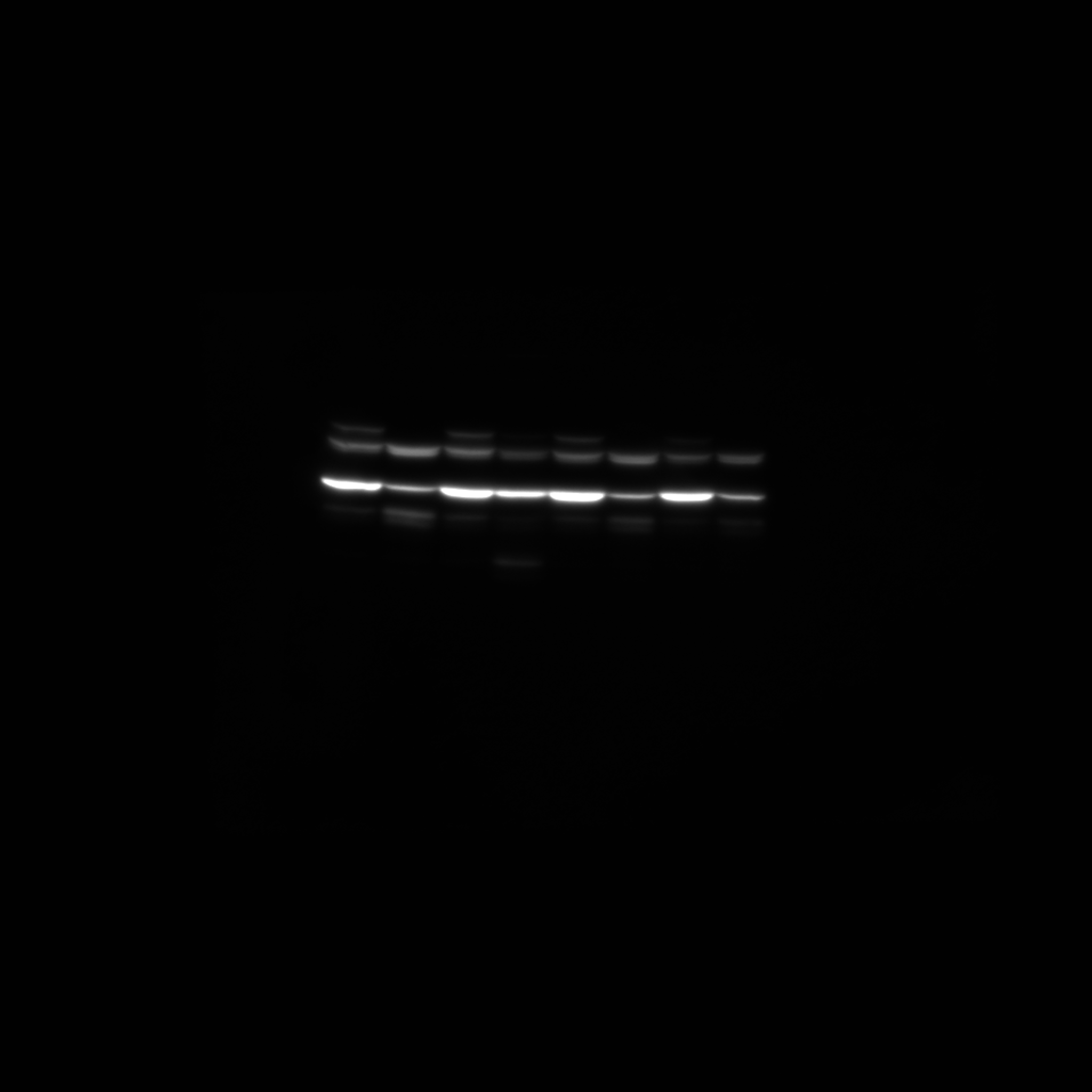

Supplement: Supplementary file 9 — EV Figure Source Data [file 44318_2024_272_MOESM9_ESM.zip › SourceData_allEV/FigureEV4/C/20240722-mitophagy flux/1-actin.Tif]

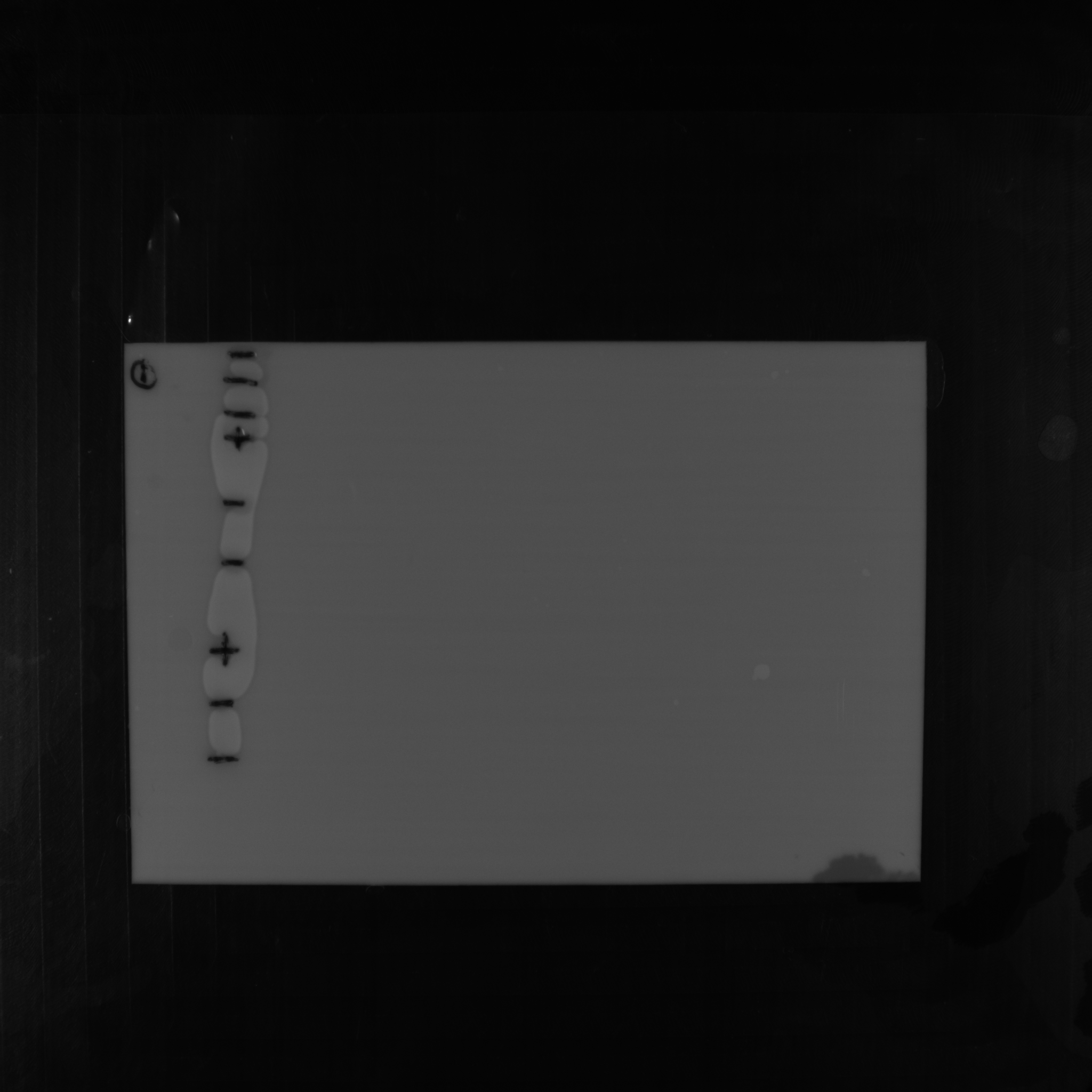

Supplement: Supplementary file 9 — EV Figure Source Data [file 44318_2024_272_MOESM9_ESM.zip › SourceData_allEV/FigureEV4/C/20240722-mitophagy flux/1-marker.Tif]

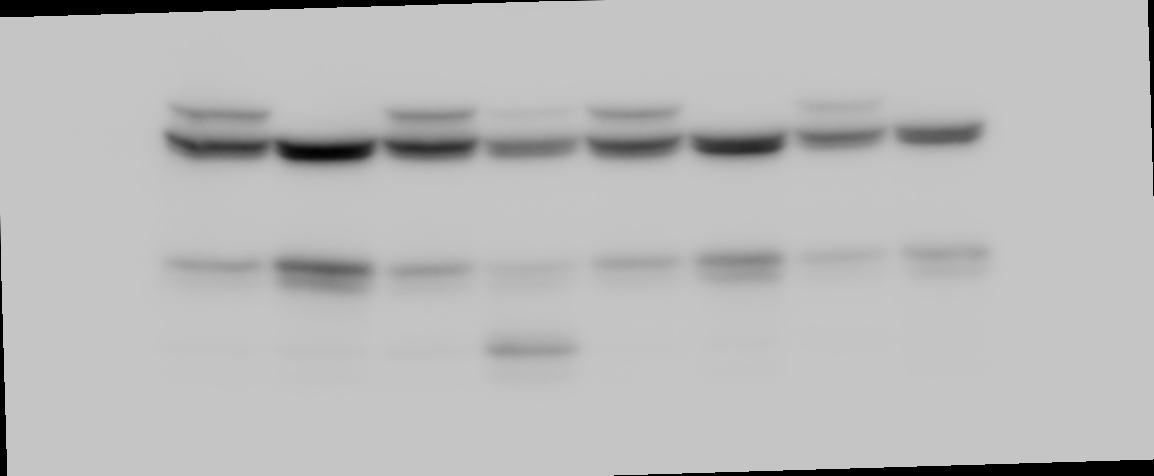

Supplement: Supplementary file 9 — EV Figure Source Data [file 44318_2024_272_MOESM9_ESM.zip › SourceData_allEV/FigureEV4/C/20240722-mitophagy flux/1-mito flux-1.tif]

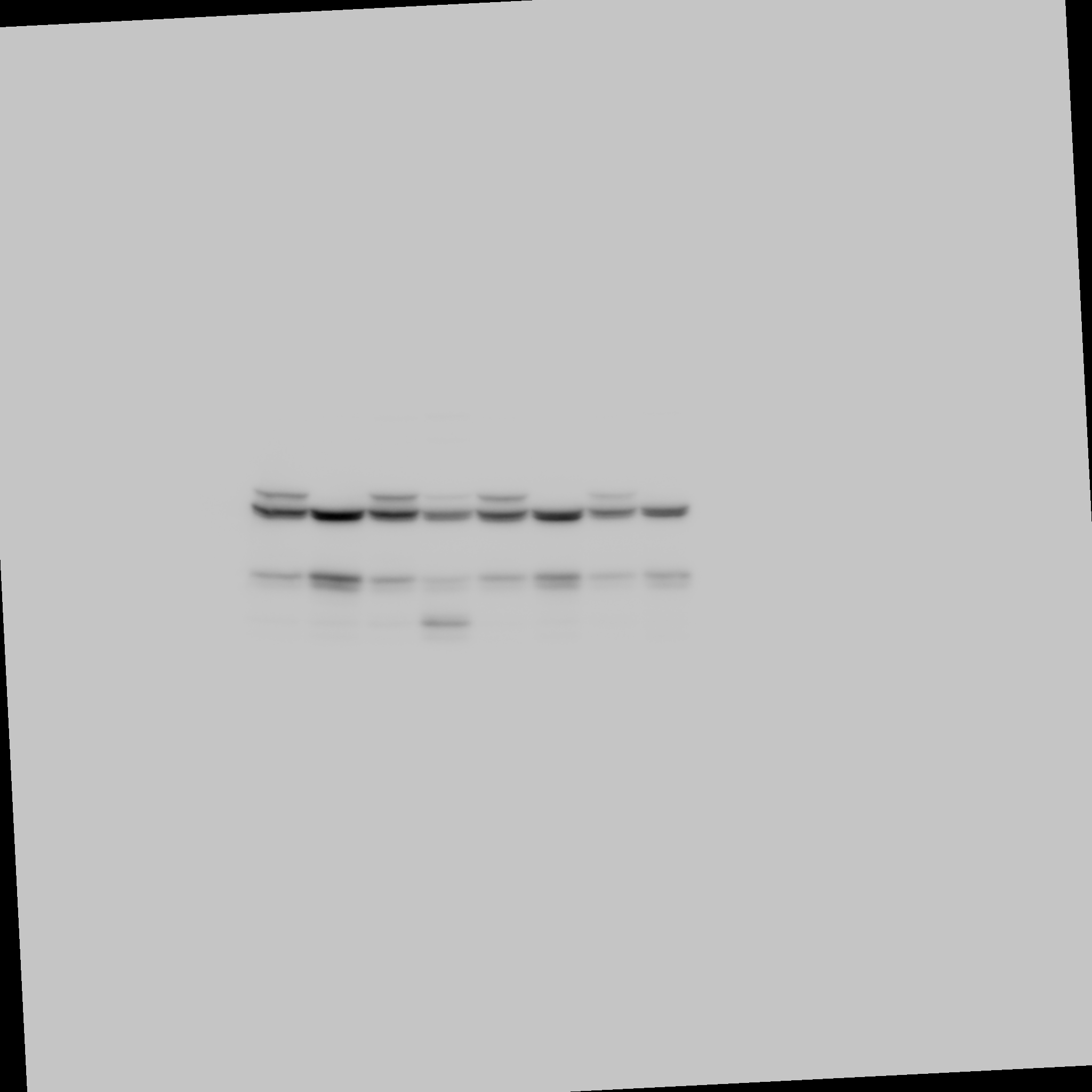

Supplement: Supplementary file 9 — EV Figure Source Data [file 44318_2024_272_MOESM9_ESM.zip › SourceData_allEV/FigureEV4/C/20240722-mitophagy flux/1-mito flux.Tif]

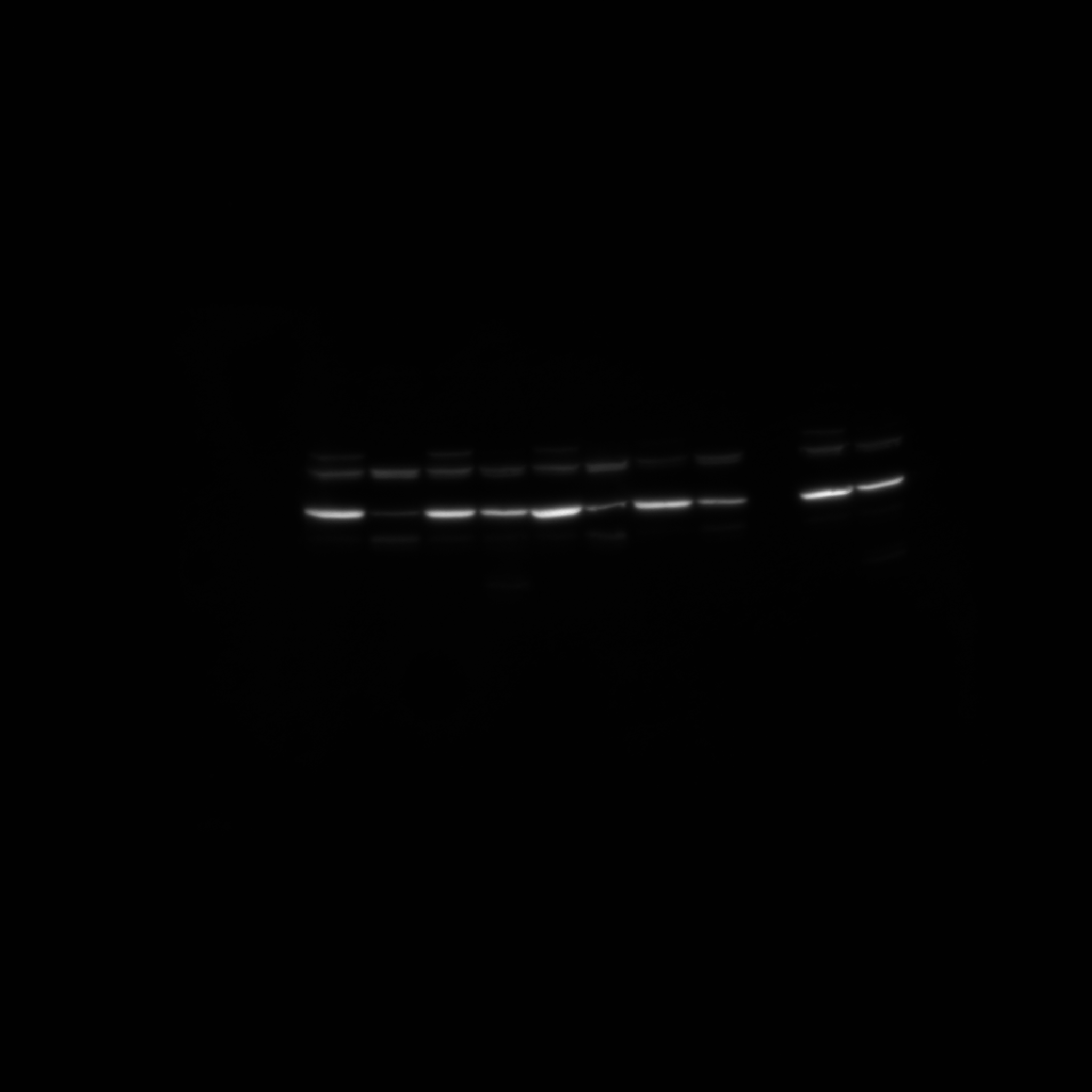

Supplement: Supplementary file 9 — EV Figure Source Data [file 44318_2024_272_MOESM9_ESM.zip › SourceData_allEV/FigureEV4/C/20240722-mitophagy flux/2-actin.Tif]

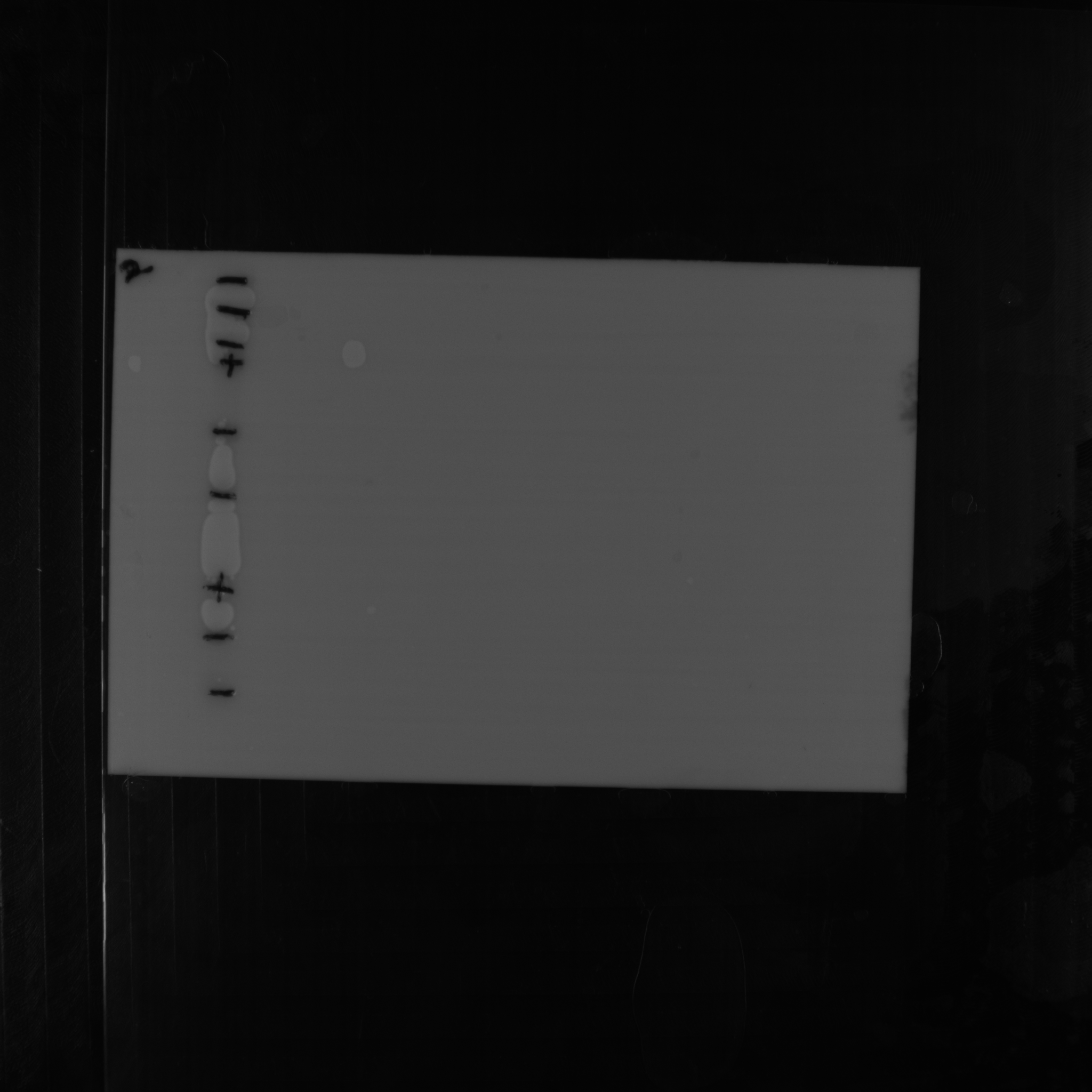

Supplement: Supplementary file 9 — EV Figure Source Data [file 44318_2024_272_MOESM9_ESM.zip › SourceData_allEV/FigureEV4/C/20240722-mitophagy flux/2-marker.Tif]

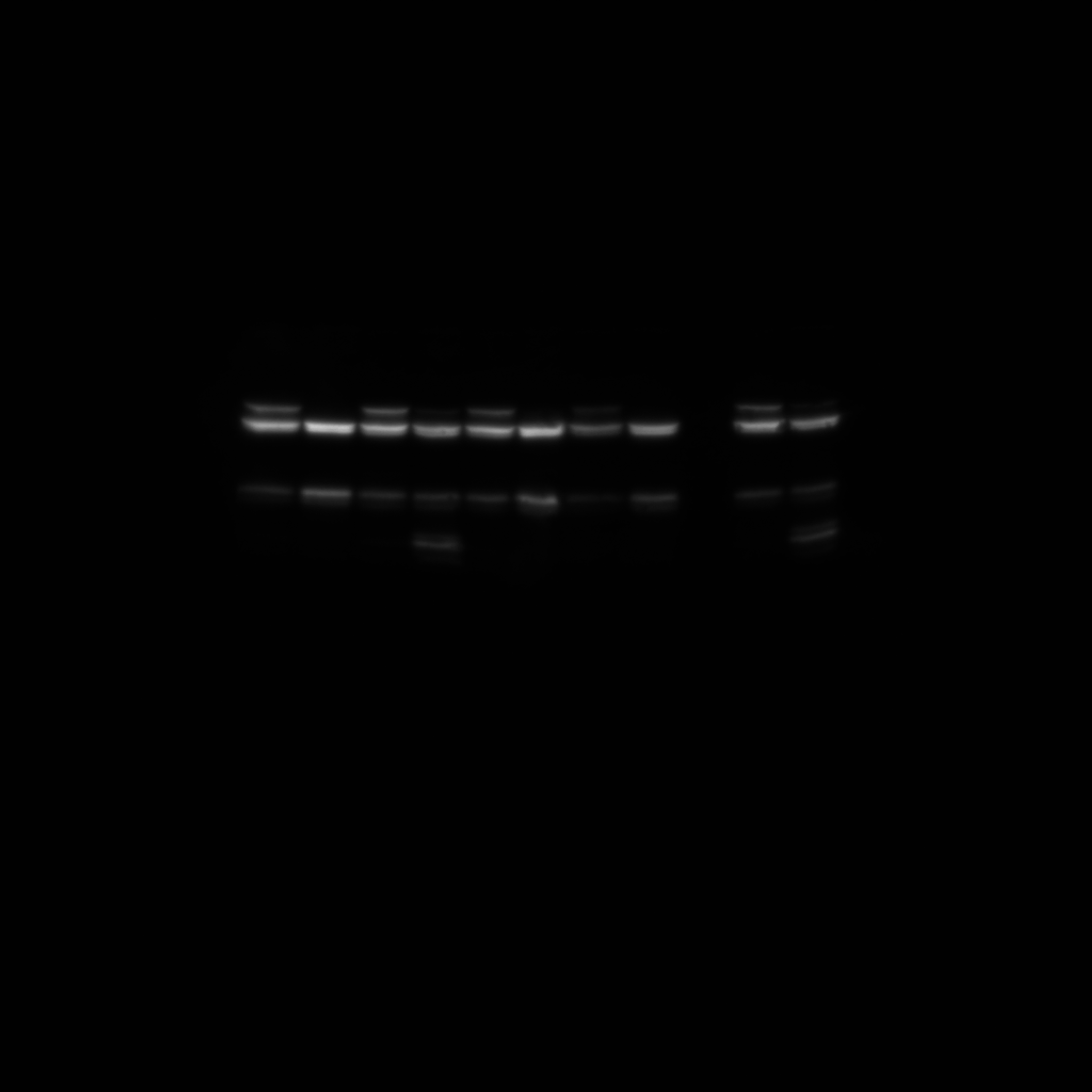

Supplement: Supplementary file 9 — EV Figure Source Data [file 44318_2024_272_MOESM9_ESM.zip › SourceData_allEV/FigureEV4/C/20240722-mitophagy flux/2-mito flux.Tif]

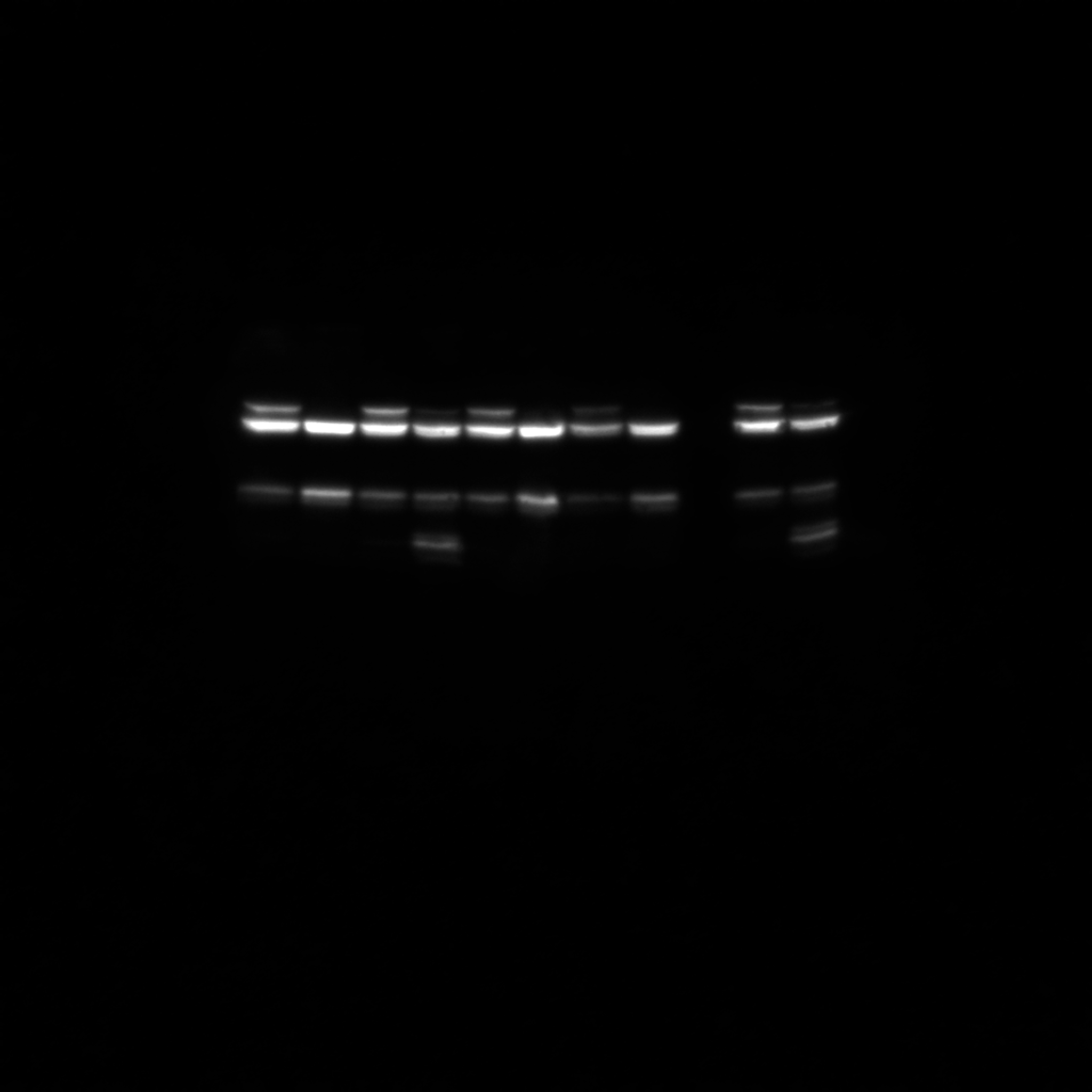

Supplement: Supplementary file 9 — EV Figure Source Data [file 44318_2024_272_MOESM9_ESM.zip › SourceData_allEV/FigureEV4/C/20240722-mitophagy flux/2-mito-modi.Tif]

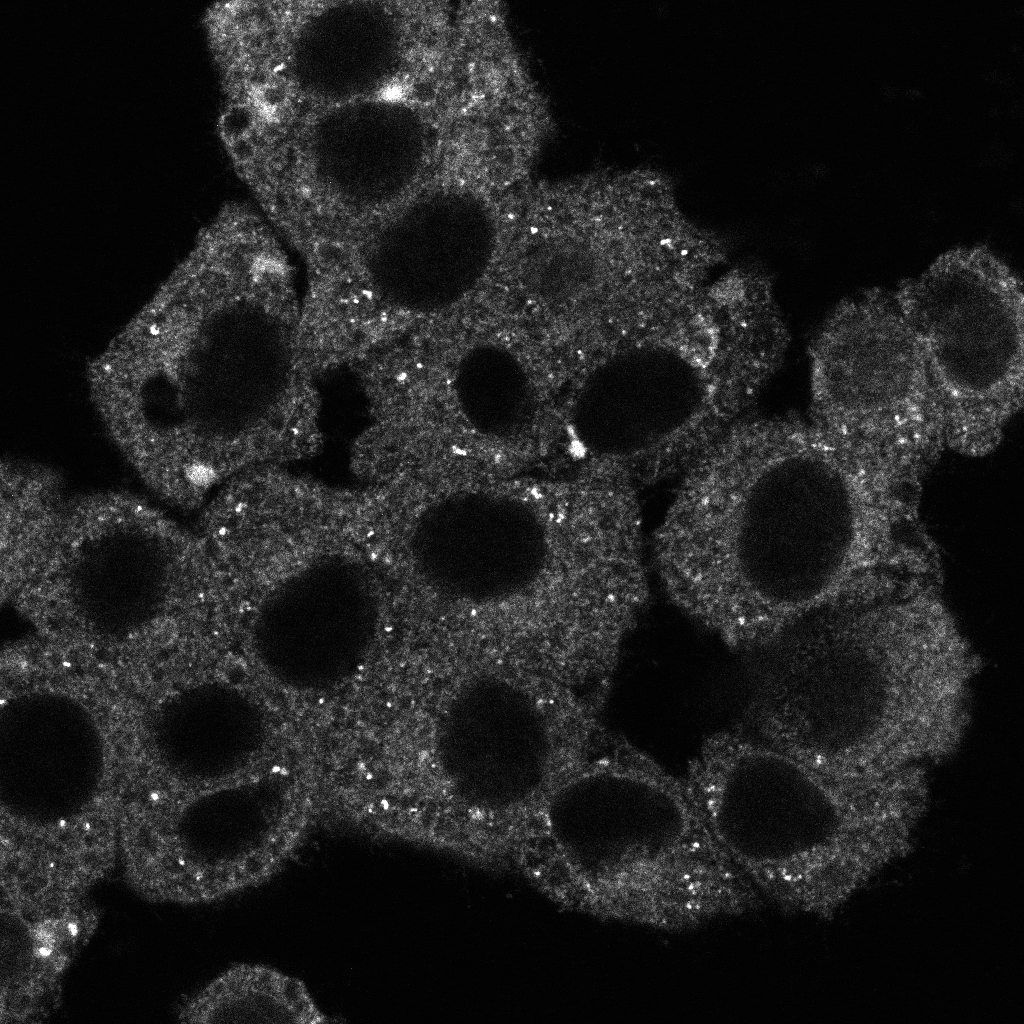

Supplement: Supplementary file 9 — EV Figure Source Data [file 44318_2024_272_MOESM9_ESM.zip › SourceData_allEV/FigureEV5/A/FIP200-OPTN+Ub.tif]

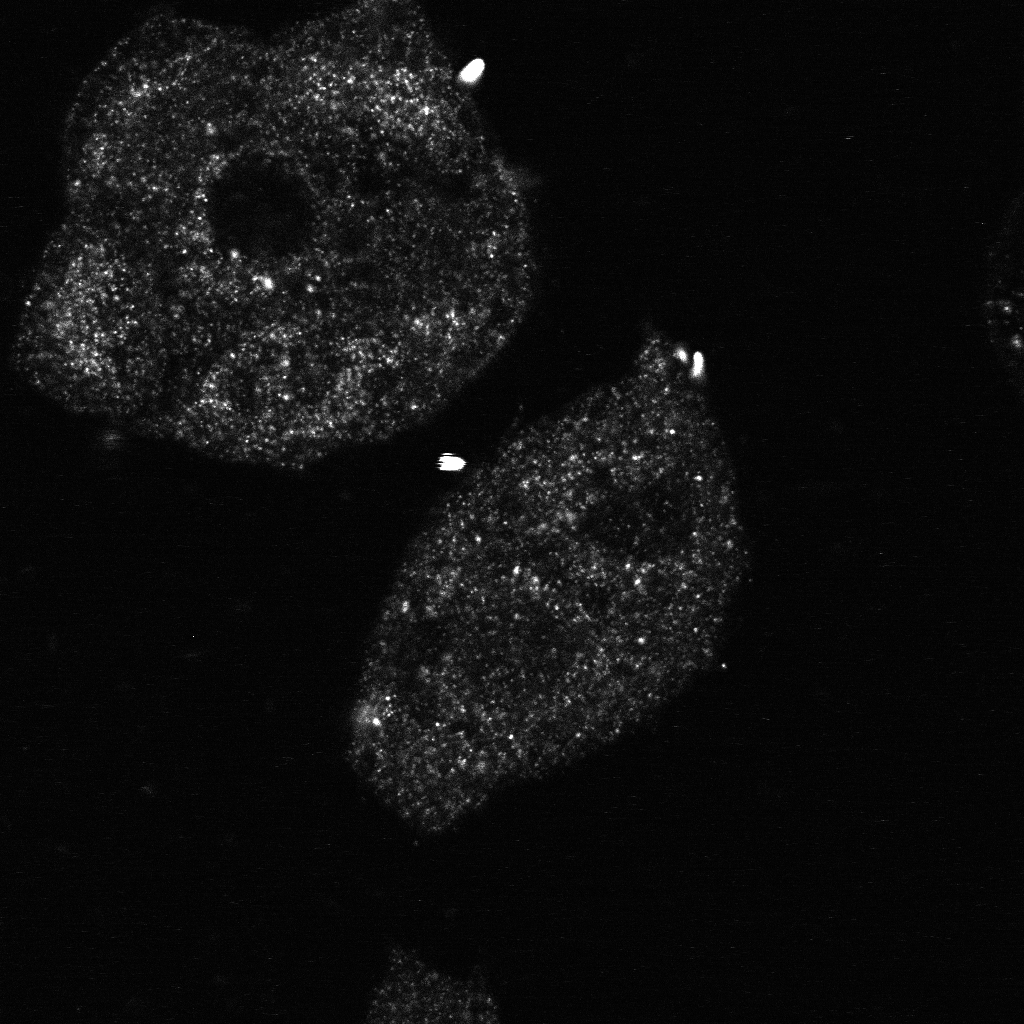

Supplement: Supplementary file 9 — EV Figure Source Data [file 44318_2024_272_MOESM9_ESM.zip › SourceData_allEV/FigureEV5/A/FIP200-OPTN-UBD-del+Nano-Ub.tif]

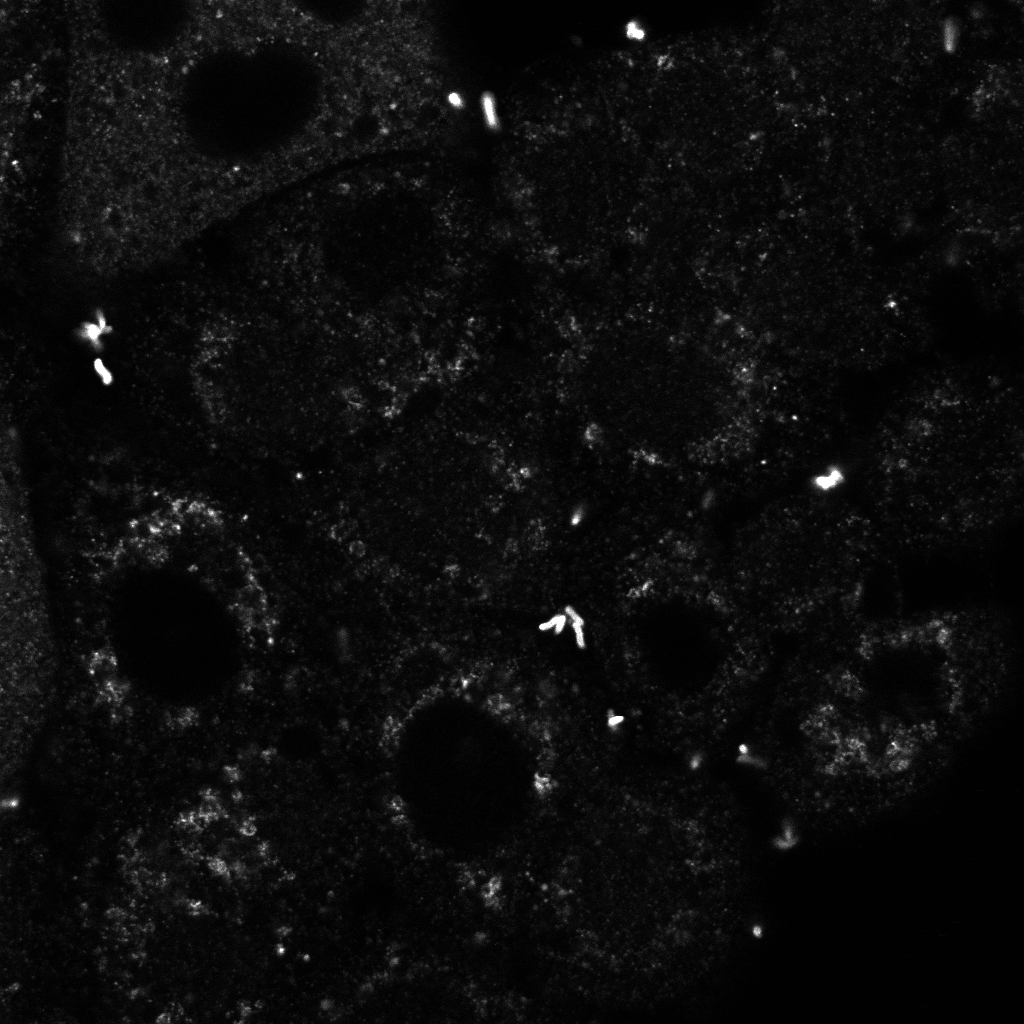

Supplement: Supplementary file 9 — EV Figure Source Data [file 44318_2024_272_MOESM9_ESM.zip › SourceData_allEV/FigureEV5/B/TBK1-OPTN+Ub.tif]

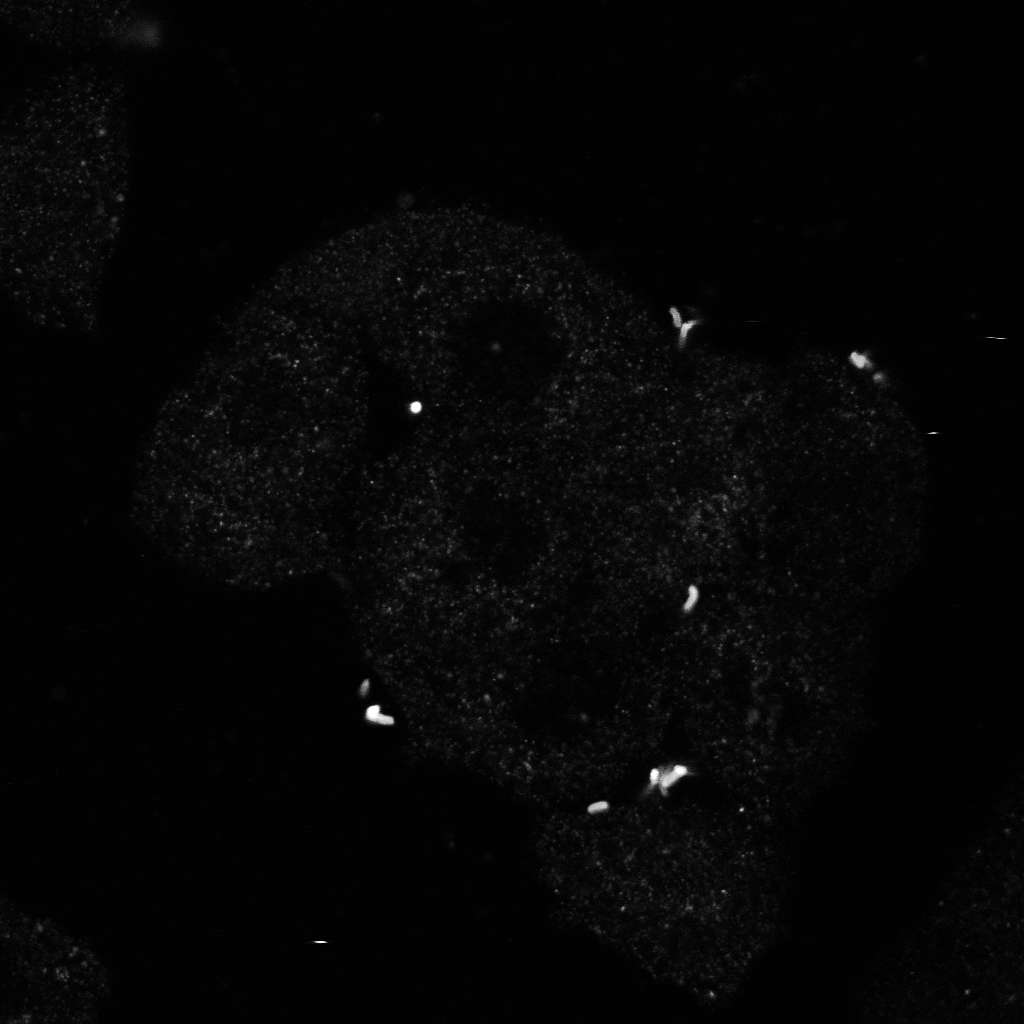

Supplement: Supplementary file 9 — EV Figure Source Data [file 44318_2024_272_MOESM9_ESM.zip › SourceData_allEV/FigureEV5/B/TBK1-OPTN-UBD-del+Nano.tif]
