# Supplementary material for: Autophagy adaptors mediate Parkin-dependent mitophagy by forming sheet-like liquid condensates
Source: EMBO J. 2024 Oct 17;43(22):5613–34. doi: 10.1038/s44318-024-00272-5 (PMC11574277; doi:10.1038/s44318-024-00272-5)
Supplement: Supplementary file 10 — Table EV1 [file 44318_2024_272_MOESM10_ESM.pdf]

Table EV1. Sequences of the primers used to construct the indicated plasmids.

Plasmid	Primer sequence	Purpose
pMRX-IPU-muGFP-(MCS)	CCGGATCTGCCATCGATATAAAGCTTGCCACCATGGTGAGCAAGGGCGAGGAGCTG	Generation of EGFP (A206K) point mutant
	GCTTTACTCGAGACCCGAATTCCCGGATCCCGAGCCTGAACCCTTGTACAGCTCGTCCATGCCatgAGTG	
pMRX-IPU-muGFP-Ubiquitin	GGTTCAGGCTCGGGATCCGGGAATTCGCAGATTTTCGTGAAAACCCTTAC	Amplification of human Ub
	GGAATTTACGTAGCGGCCGCTCTCGAGTTAACCACCACGAAGTCTCAAC	
pMRX-IPU-muGFP-p62	GGTTCAGGCTCGGGATCCGGGAATTCGGCGTCGCTCACCGTGAAGGCC	Amplification of human p62
	TTTACGTAGCGGCCGCTCTCGAGTCACAACGGCGGGGGATGCTTTG	
pMRX-IPU-muGFP-OPTN	GGTTCAGGCTCGGGATCCGGGAATTCGTCCCATCAACCTCTCAGCTGCCTCACTG	Amplification of human OPTN
	GGAATTTACGTAGCGGCCGCTTTACTCGAGACCTTAAATGATGCAATCCATCACGTGAATC	
pMRX-IPU-muGFP-NBR1	GGTTCAGGCTCGGGATCCGGGAATTCGGAACCACAGGTTACTCTAAATGTGAC	Amplification of human NBR1
	GGAATTTACGTAGCGGCCGCTTTACTCGAGACCTCAATAGCGTTGGCTGTACCAGTCG	
pMRX-IPU-muGFP-NDP52	GGTTCAGGCTCGGGATCCGGGAATTCGGAGGAGACCATCAAAGATCCCCC	Amplification of human NDP52
	GGAATTTACGTAGCGGCCGCTTTACTCGAGACCTCAGAGAGAGTGGCAGAACACGTGG	
pMRX-IPU-muGFP-TAX1BP1	GGTTCAGGCTCGGGATCCGGGAATTCGACATCCTTTCAAGAAGTCCC	Amplification of human TAX1BP1
	GGAATTTACGTAGCGGCCGCTTTACTCGAGCTAGTCAAAATTTAGAACATTCTG	
pMRX-IHU-mRuby-nano-ubiquitin	AGGCTCGGGATCCGGGAATTCGGCGCAGGTTCAGCTGGTTGAAAGCGGTG	Amplification of GFP-nanobody
	GCTGCCGCTTCCGCTACCTTTGCTGCTAACGGTAACCTGGGTGCCCTGAC	
	GGTAGCGGAAGCGGCAGCCAGATTTTCGTGAAAACCCTTACGGGGAAGAC	Amplification of ubiquitin
	GTAGCGGCCGCTCTCGAGACCTTAACCACCACGAAGTCTCAACACAAG	
pMRX-IPU-GFP-OPTNΔUBAN	CCAAACAGCTGCAAATGAATGATGCTTTCGAAGACGGAGGCAGGC	Generation of OPTN (ΔUBAN) deletion mutant
	CTTCGAAAGCATCATTCATTTGCAGCTGTTTGGAAGCCAGAGCCTTC	
pMRX-IBU-Su9-HaloTag7-SNAP	CGATATAAAGCTTAAAGGATCCATGGCCTCCACTCGTGTCCTCGCCTC	Amplification of *N. crassa* F_0-ATPase subunit 9
	GCTGCCGCTGCCGCTTCCGCTACCGGTGGCGACCGGTGGATCAGAAGAG	
	GCGGAAGCGGCAGCGGCAGCGGCGAAATCGGTACTGGCTTTCCATTCG	Amplification of HaloTag7
	GCCTGAACCAGAGCCCGAATTCCCACCGGAAATCTCCAGAGTAGACAGC	
pMRX-IPU-EGFP-ATG9A	GGCCGGATCCGCGCAGTTTGACACTGAATACC	Amplification of human ATG9A
	GGCCGAATTCTTACTATACCTTGTGCACCTGAGGGGG	
pMRX-IPU-GFP-	CAGCGAGAGA GAAAATTCAT	Generation of OPTN (E478G)
	GAATTTTCTCTCTCGCTGCTCTTCCAGC ATGAAAATCA G	

OPTN(E478G)		mutant
pMRX-IPU-OPTNΔUBAN-GFP	GATCTGCCATCGATATAAAGATGTCCCATCAACCTCTCAGCTGCCTC	Generation of OPTN (ΔUBAN) deletion mutant
	CTTGCTCACCATGGTGGCAAGGGATCCCGAGCCTGAACCAATGATGCAATCCATCACGTGAATCTGTAACGTGTC	
