# Supplementary material for: Autophagy adaptors mediate Parkin-dependent mitophagy by forming sheet-like liquid condensates
Source: EMBO J. 2024 Oct 17;43(22):5613–34. doi: 10.1038/s44318-024-00272-5 (PMC11574277; doi:10.1038/s44318-024-00272-5)
Supplement: Supplementary file 11 — Expanded View Figures [file 44318_2024_272_MOESM11_ESM.pdf]

Expanded View Figures

Figure EV1. Localisation of autophagy adaptors between mitochondria during Parkin-mediated mitophagy.

(**A**) Representative images (left) and spline graphs of the intensity profiles along the indicated arrows (from position 0; right) of wild-type HeLa cells expressing one of the GFP-tagged autophagy adaptors or ubiquitin and mRuby–Omp25 at 45 min after CCCP treatment. Mitochondrial clusters (Mt–Mt) are shown. The *y*-axis in each of the graphs indicates the fluorescence intensity. (**B**) The relative intensity of each adaptor was calculated as in Fig. 1C. Solid horizontal bars indicate the means, and dots indicate the data from five structures. Differences were statistically analyzed by one-way ANOVA with Dunnett's post-hoc test. (**C**) Representative images (left) and spline graphs of the intensity profiles along the indicated arrows (from position 0; right) of HeLa cells lacking all five autophagy adaptors (penta KO cells) expressing one of the GFP-tagged autophagy adaptors or ubiquitin and mRuby–Omp25 at 45 min after CCCP treatment. Mitochondrial clusters (Mt–Mt) are shown. The *y*-axis in each of the graphs indicates the fluorescence intensity. (**D**) The relative intensity of each adaptor was calculated as in Fig. 1C. Solid horizontal bars indicate the means, and dots indicate the data from five structures. Differences were statistically analyzed by one-way ANOVA with Dunnett's post-hoc test. Source data are available online for this figure.

© The Author(s)

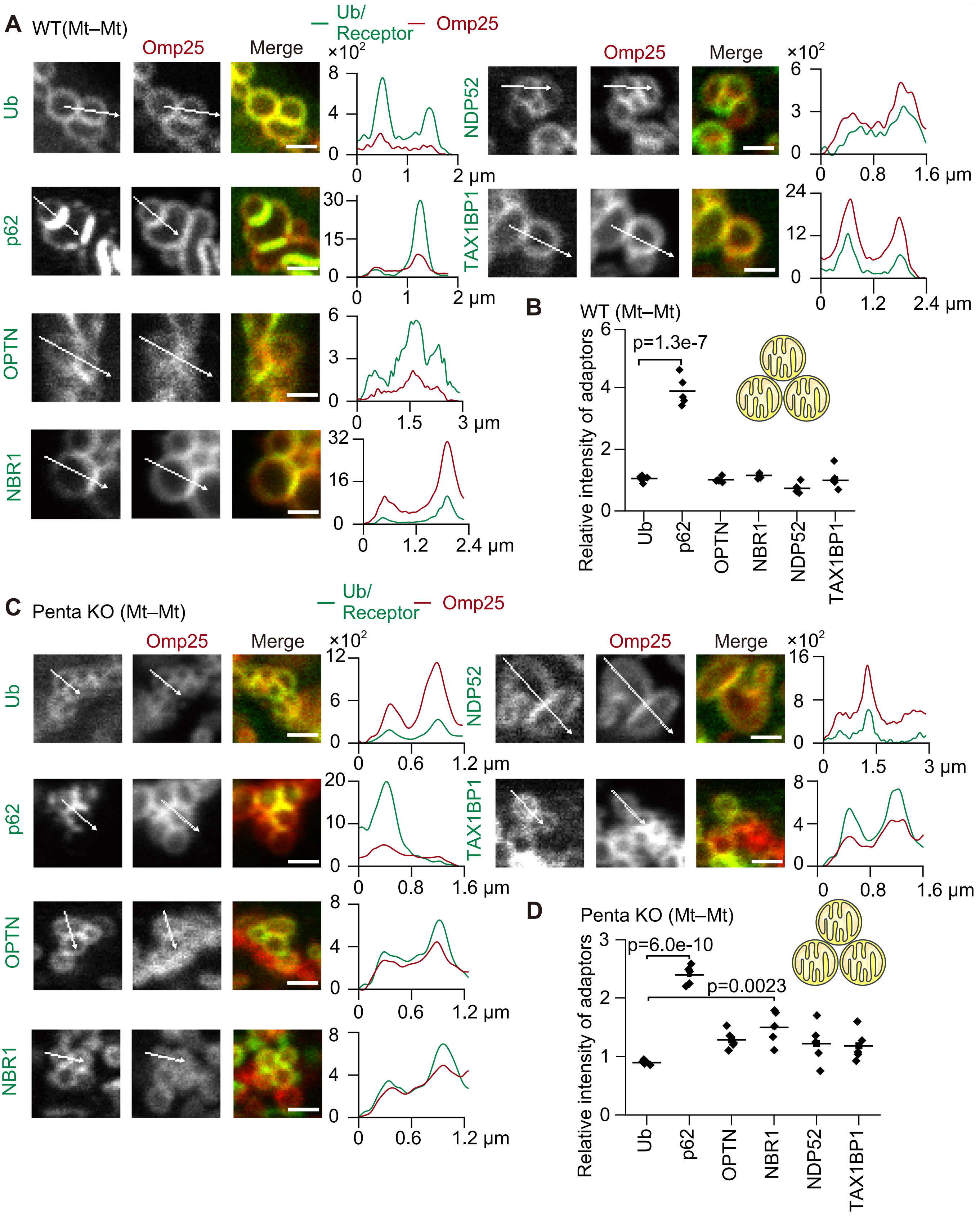
A WT(Mt–Mt)
Ub/ Receptor
Omp25
Merge
×10²
Ub
p62
OPTN
NBR1
NDP52
TAX1BP1
µm
B
WT (Mt–Mt)
p=1.3e-7
Relative intensity of adaptors
C Penta KO (Mt–Mt)
D
Penta KO (Mt–Mt)
p=6.0e-10
p=0.0023

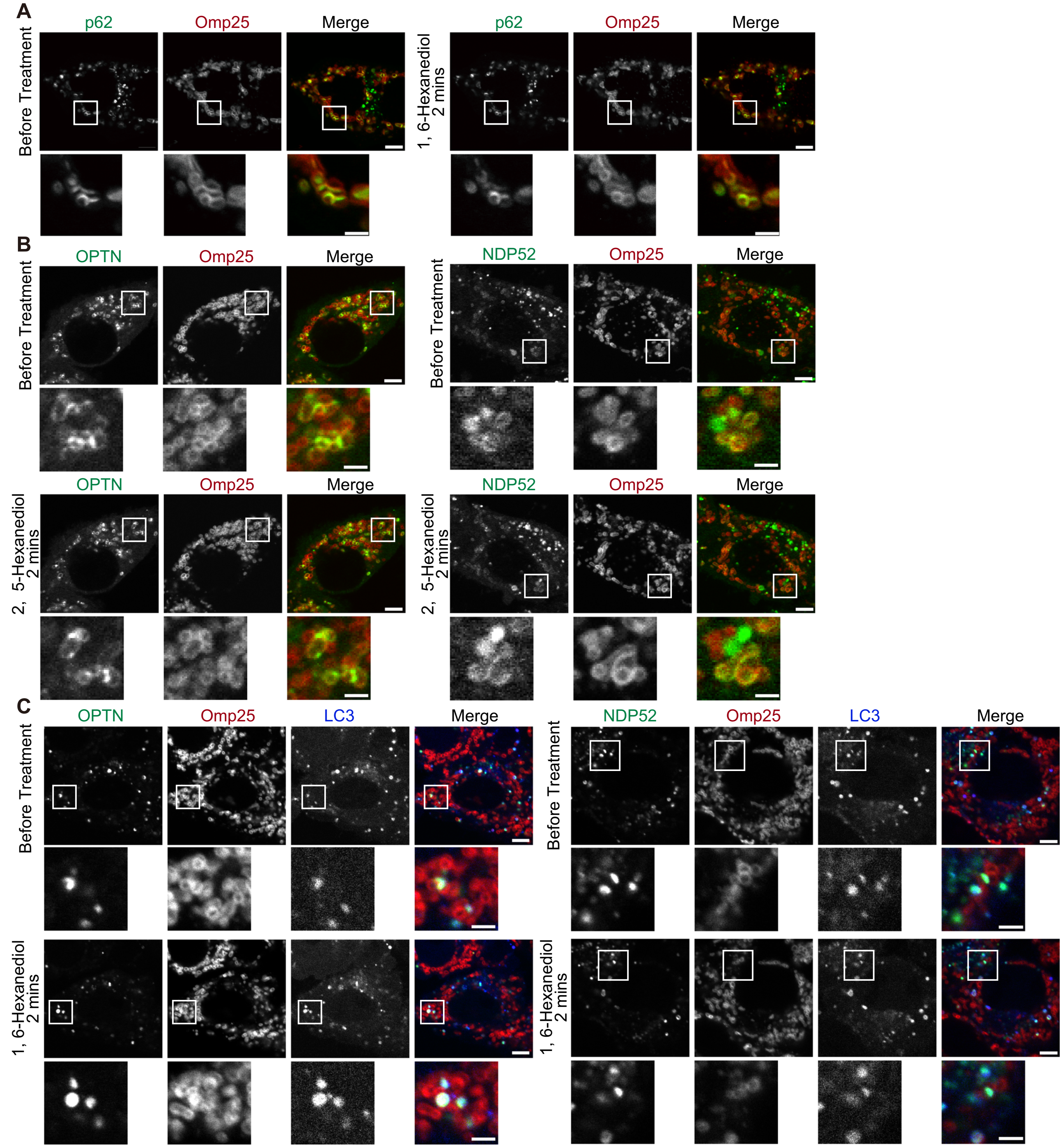

Figure EV2. p62 on mitochondrial clusters and OPTN and NDP52 at the mitochondria-isolation membrane contact sites are resistant to 1,6-hexanediol treatment.

(**A**) Penta KO cells expressing GFP-p62 at 45 min after CCCP treatment. Images of cells before (left) and 2 min after (right) the addition of 10% 1,6-hexanediol are displayed. Scale bars, 5 and 2 μm (magnified images). (**B**) Penta KO cells expressing GFP-OPTN (left) or GFP-NDP52 (right) at 45 min after CCCP treatment. Images of cells before (upper panels) and 2 min after (lower panels) the addition of 10% 2,5-hexanediol are displayed. Scale bars, 5 and 2 μm (magnified images). (**C**) Penta KO cells expressing GFP-OPTN (left) or GFP-NDP52 (right) at 45 min after CCCP treatment. Mitochondria associated with isolation membranes (LC3) were analyzed. Images of cells before (upper panels) and 2 min after (lower panels) the addition of 10% 1,6-hexanediol are displayed. Scale bars, 5 and 2 μm (magnified images). Source data are available online for this figure.

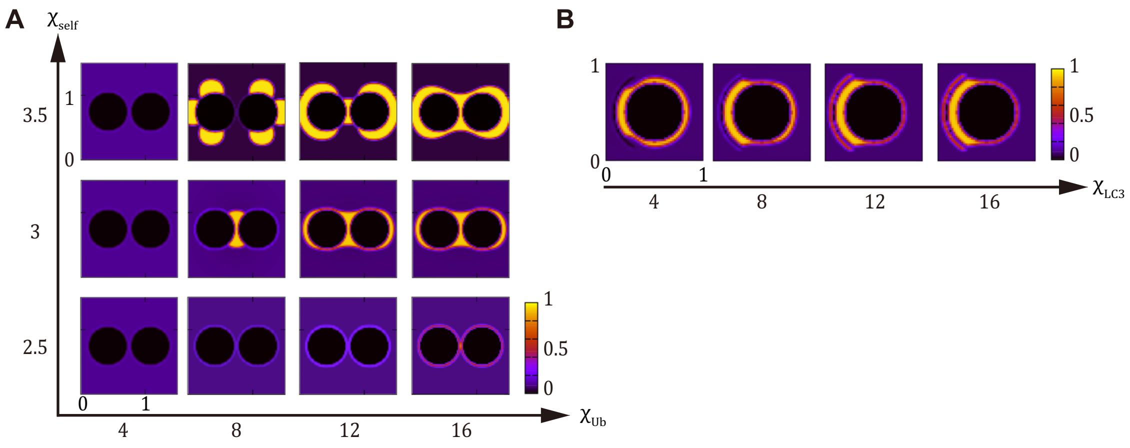

Figure EV3. Droplet distribution depends on the binding strength.

(**A**) Dependence of droplet distribution on two mitochondria on Ub-binding strength (χ_{Ub}) and self-binding strength (χ_{self}). Mitochondrial area exclusion (χ_{Mt}) and surface tension (σ) were set to $\chi_{Mt} = 5k_BT$ and $\sigma = k_BT$, respectively. (**B**) Dependence of droplet distribution on isolation membrane and mitochondria on LC3-binding strength (χ_{LC3}). The other parameters were set to $\chi_{Mt} = 5k_BT$, $\sigma = k_BT$, $\chi_{self} = 3k_BT$ and $\chi_{Ub} = 12k_BT$. The bending angle of the isolation membrane was set to $\alpha = \pi/3$.

 © The Author(s)

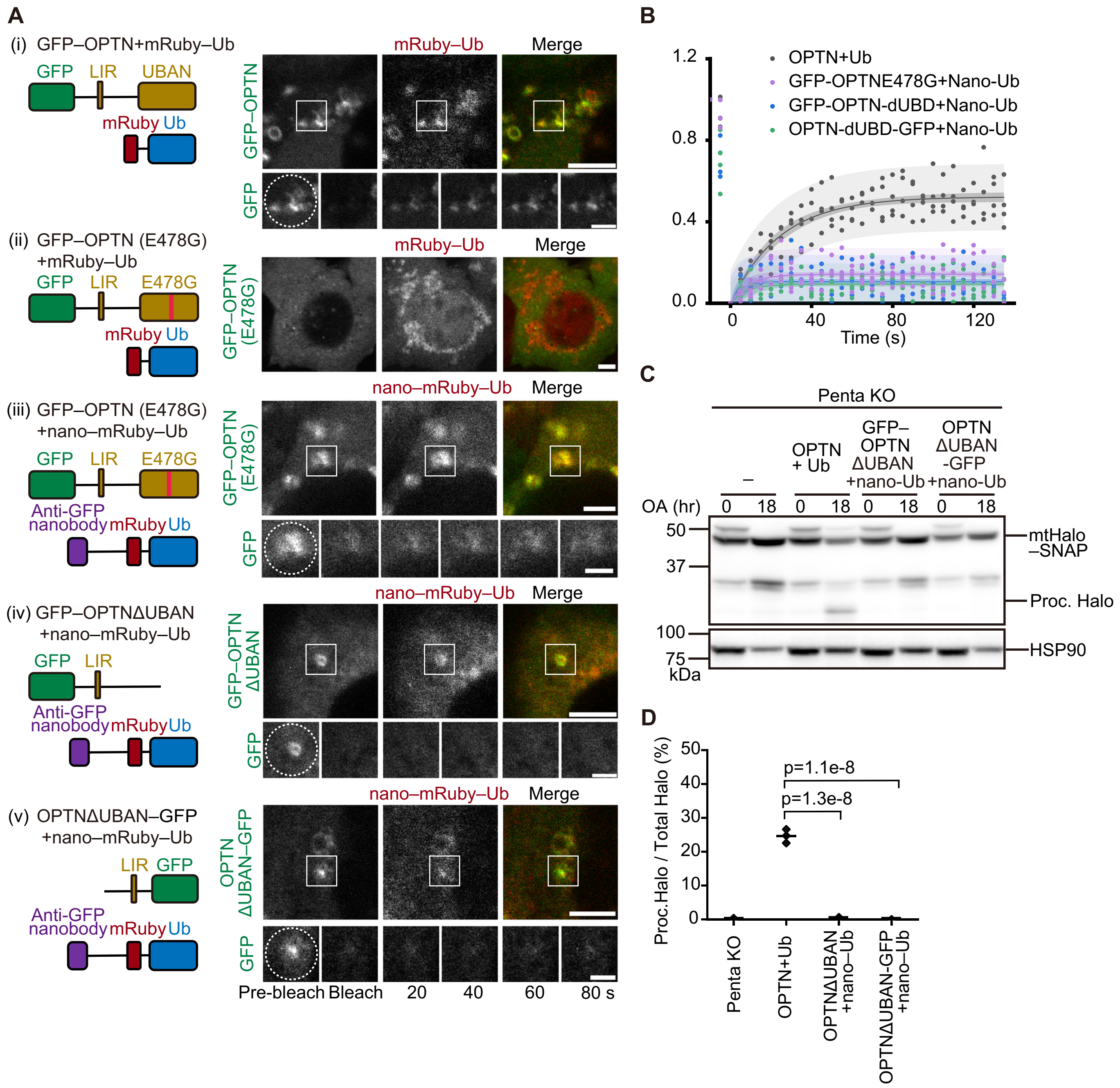

Figure EV4. Loss of mobility and mitophagy activity with ubiquitin binding mutants of OPTN.

(**A**) Penta KO cells expressing both GFP-OPTN and mRuby-Ub (i), GPF-OPTN (E478G) and mRuby-Ub (ii), both GPF-OPTN (E478G) and anti-GFP nanobody-mRuby-Ub (iii), both GFP-OPTNΔUBAN and anti-GFP nanobody-mRuby-Ub (iv), and both OPTNΔUBAN-GFP and anti-GFP nanobody-mRuby-Ub (v) at 45 min after CCCP treatment. Time-lapse images of GFP FRAP are shown. Photobleached areas are circled by dotted lines. Scale bars indicate 4 and 2 μm (magnified images). (**B**) Quantification of GFP FRAP on separate mitochondria (Mt) in penta KO cells expressing both GFP-OPTN and mRuby-Ub (i), both GPF-OPTN (E478G) and anti-GFP nanobody-mRuby-Ub (iii), both GFP-OPTNΔUBAN and anti-GFP nanobody-mRuby-Ub (iv), and both OPTNΔUBAN-GFP and anti-GFP nanobody-mRuby-Ub (v) at 45 min after CCCP treatment. Data were collected from four structures and were fitted to the equation $y = a*(1 - \exp(-b*x))$. The dark shading represents the 95% confidence intervals, and the light shading represents the 95% prediction intervals. (**C**, **D**) Representative data (**C**) and quantification (**D**) of HaloTag (Halo) processing assay using cells expressing the indicated OPTN and Ub constructs. Cells expressing the mtHalo-SNAP mitophagy reporter were treated without (0 h) and with oligomycin and antimycin for 18 h. The amount of processed Halo (proc. Halo) indicates the relative amount of mitochondria degraded in lysosomes. Solid horizontal bars indicate the means, and dots indicate the data from three independent cultures. Differences were statistically analyzed by one-way analysis of variance with Dunnett's post-hoc test. Source data are available online for this figure.

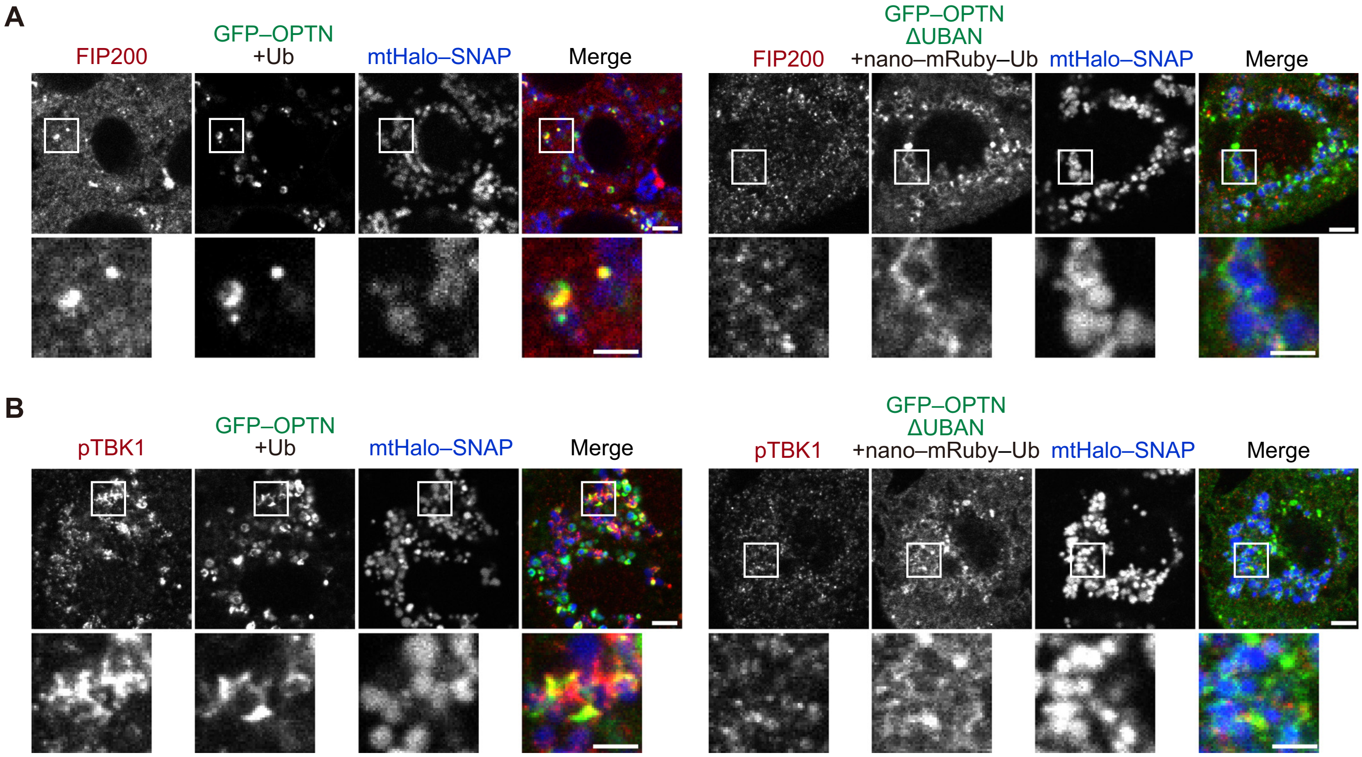

Figure EV5. Recruitment of TBK1 and FIP200 during mitophagy.

(**A**, **B**) Localisation of FIP200 and phosphorylated TBK1 under mitophagy-inducing conditions (CCCP, 60 min). Endogenous FIP200 (**A**) and phosphorylated TBK1 (**B**) were immunostained in penta KO cells expressing both GFP-OPTN and mRuby-Ub or both GFP-OPTNΔUBAN and anti-GFP nanobody-mRuby-Ub together with mitochondrially targeted Halo-SNAP (mtHalo-SNAP). Scale bars indicate 4 and 2 μm (magnified images). Source data are available online for this figure.

 © The Author(s)
